# Reorganization of 3D genome structure may contribute to gene regulatory evolution in primates

**DOI:** 10.1371/journal.pgen.1008278

**Published:** 2019-07-19

**Authors:** Ittai E. Eres, Kaixuan Luo, Chiaowen Joyce Hsiao, Lauren E. Blake, Yoav Gilad

**Affiliations:** 1 Department of Human Genetics, University of Chicago, Chicago, Illinois, United States of America; 2 Department of Medicine, University of Chicago, Chicago, Illinois, United States of America; Fred Hutchinson Cancer Research Center, UNITED STATES

## Abstract

A growing body of evidence supports the notion that variation in gene regulation plays a crucial role in both speciation and adaptation. However, a comprehensive functional understanding of the mechanisms underlying regulatory evolution remains elusive. In primates, one of the crucial missing pieces of information towards a better understanding of regulatory evolution is a comparative annotation of interactions between distal regulatory elements and promoters. Chromatin conformation capture technologies have enabled genome-wide quantifications of such distal 3D interactions. However, relatively little comparative research in primates has been done using such technologies. To address this gap, we used Hi-C to characterize 3D chromatin interactions in induced pluripotent stem cells (iPSCs) from humans and chimpanzees. We also used RNA-seq to collect gene expression data from the same lines. We generally observed that lower-order, pairwise 3D genomic interactions are conserved in humans and chimpanzees, but higher order genomic structures, such as topologically associating domains (TADs), are not as conserved. Inter-species differences in 3D genomic interactions are often associated with gene expression differences between the species. To provide additional functional context to our observations, we considered previously published chromatin data from human stem cells. We found that inter-species differences in 3D genomic interactions, which are also associated with gene expression differences between the species, are enriched for both active and repressive marks. Overall, our data demonstrate that, as expected, an understanding of 3D genome reorganization is key to explaining regulatory evolution.

## Author summary

The way in which a genome folds affects the regulation of gene expression. This is often due to loops in the three-dimensional structure that bring linearly distant genes and regulatory elements into close proximity. Most studies examining three-dimensional structure genome-wide are limited to a single species. In this study, we compared three-dimensional structure in the genomes of induced pluripotent stem cells from humans and chimpanzees. We collected gene expression data from the same samples, which allowed us to assess the contribution of three-dimensional chromatin conformation to gene regulatory evolution in primates. Our results demonstrate that gene expression differences between the species may often be mediated by differences in three-dimensional genomic interactions. Our data also suggest that large-scale chromatin structures (i.e. topologically associating domains, TADs) are not well conserved in their placement across species. We hope the analytical paradigms we present here could serve as a basis for future comparative studies of three-dimensional genome organization, elucidating the putative functional regulatory loci driving speciation.

## Introduction

A growing body of evidence indicates that variation in gene regulation plays a key role in phenotypic divergence between species [1–7]. Inferring the causal relationship between inter-species regulatory differences and phenotypic differences between species remains challenging, but compelling examples of regulatory adaptations have been published in a large number of species, including primates [8–13]. The molecular mechanisms that underlie regulatory adaptation have also been the focus of much research. Studies in mice, flies, yeast, and primates have revealed that expression divergence between species is often driven by mutations or epigenetic modifications within cis-regulatory elements (CREs), rather than *trans* elements (e.g. transcription factors [5, 8–12, 14–15]). This makes intuitive sense, because transcription factors can operate broadly across multiple functional contexts and throughout the genome (affecting many genes), whereas CREs often have more specific functional outcomes [3, 16].

The ability to measure epigenetic marks, chromatin structure, and other functional genomic data has enabled us to identify and classify CREs into different types of regulators with distinct effects on gene expression (e.g. enhancers, silencers, insulators) [17–19]. Despite significant advances in our ability to identify and predict the functional role of CREs, we still lack a comprehensive characterization of the functional relationships between CREs and the genes they regulate. In many cases, we still do not know which genes are regulated by which CREs, or when and how often these relationships change. Connecting CREs to their target genes is crucial for understanding how regulatory architecture changes in response to different spatial, temporal and organismal contexts [15, 17–22]. Ultimately, the effects of CREs on gene expression are likely to depend on which promoter(s) they contact, which is inherently related to the 3D structure of the genome [23, 24].

The proximity and frequency of CRE-gene contacts can be measured *in vivo* using chromosome conformation capture techniques [25]. Chromosome conformation affects how genes are expressed within a cell [26–33]. For example, 3D genome structures may bring linearly distant loci into close proximity, connecting genes with CREs [34–40]. Expressed genes have been observed to spatially localize with distant CREs in 3D FISH experiments [35, 41]. The latest chromosome conformation capture based technique, Hi-C, pairs the original method’s proximity-based ligation with high-throughput sequencing to identify DNA-DNA contacts on a genome-wide scale [42]. With enough sequencing coverage, Hi-C data can ultimately yield a comprehensive map of the 3D structure of an entire genome at high resolution [43].

Divergence in 3D genome structure may lead to regulatory evolution and ultimately to adaptation of new phenotypes. Currently, however, there are only a small number of comparative Hi-C data sets that can be used to test this notion [44–46], and even fewer comparative data sets in primates [47, 48]. Most Hi-C studies to date have focused primarily on variation in chromatin contact frequencies within a single species [42, 49–51]. The few comparative Hi-C studies published to date typically draw comparisons between distantly related species (such as human and mouse [45, 46]), use cancerous or otherwise transformed cell lines [45], and rely on low resolution genome-wide Hi-C data (typically collecting 100-600M reads from most samples [44, 46, 52]). These comparative studies typically collect data in only a single individual from each species, and often compare contacts that are inferred from Hi-C libraries with large differences in read depth between species, a property that leads to differences in power to infer 3D genome structures at multiple scales [44–46].

Thus, to conduct a comparative Hi-C study in primates and address these challenges, we collected high resolution Hi-C data from iPSCs derived from four human and four chimpanzee individuals. The human and chimpanzee genomes share a high degree of synteny [53–58], thus allowing us to consider a comparison of both low and high order chromatin interactions. Using our data, we were able to characterize ‘lower-order’ locus-locus contacts and to infer ‘higher-order’ structural features, such as TADs and TAD boundaries. We also quantified gene expression levels using RNA-seq data from the same eight cell lines. We considered our data with existing functional annotations, including histone marks and chromatin accessibility data, and evaluated the extent to which inter-species variation in 3D genome structure and epigenetic profiles are associated with gene expression divergence between humans and chimpanzees.

## Results

We performed *in situ* Hi-C as previously described [45] on a sex-balanced panel of four human and four chimpanzee integration-free iPSC lines that were previously generated and quality-checked by the Gilad lab [59]. Using HiCUP [60] and HOMER [61] (see Methods), we obtained genome-wide Hi-C contact maps at 10 kb resolution for all eight individuals, with each map containing approximately one billion sequencing reads. Since there is currently no gold standard for Hi-C normalization and statistical modeling, we also used an alternative method, Juicer [62], to confirm that our results are robust with respect to the choices of normalization schemes and modeling (S1–S6 Figs). We also demonstrated the robustness of our results by performing certain analyses using different resolutions (from 10 kb to 500 kb). In the main text we report the results obtained using the HOMER pipeline at 10 kb resolution. Results using the alternative pipelines are shown in the supplement.

We used HOMER to independently classify between 779,503–883,438 contacts (*P* < 0.01) in the Hi-C data obtained from each individual (genomic coordinates of all contacts in all individuals are provided in S1–S8 Tables). We define a ‘contact’ as a pair of 10 kb regions which we observed to be in physical proximity more often than expected by chance. Throughout the paper, we refer to Hi-C contacts as ‘lower-order’ or ‘pairwise’ interactions in order to distinguish them from higher-order, chromosome-scale structures (i.e. TADs and TAD boundaries).

Our goal was to compare Hi-C contacts between humans and chimpanzees. One intuitive approach to do so might be to identify the orthologous locations of each contact in the two species and classify such contacts as shared or unshared. However, this could lead to an inflated estimate of inter-species differences due to incomplete power to identify contacts in one species or the other. Instead, we collected the coordinates of all contacts identified in at least one individual into a single database. For each contact in the database, we independently identified the pair of corresponding orthologous regions in the human and chimpanzee genomes (using reciprocal searches, to avoid bias). Using this approach, we excluded about 18% of contacts because we failed to identify clear orthologous regions in the genomes of the two species (see Methods and S9 Table). Following the orthology based filtering, we extracted the normalized contact frequencies (log2 observed:expected read count ratios) for all pairs of loci in the database, regardless of whether they were classified as contacts by HOMER. Thus, our analysis is not biased by potential differences in power to detect contacts in one species over the other. That said, we observed that the variance in contact frequency was lower for interactions that were independently identified in a greater number of samples, regardless of species (S7 Fig). We thus filtered out interactions that were independently classified as significant in fewer than four individuals. This approach allowed us to compare contact frequencies between species for 347,206 interactions while largely sidestepping the problem of incomplete power.

### Inter-species differences in 3D genomic interactions

**Fig 1:**
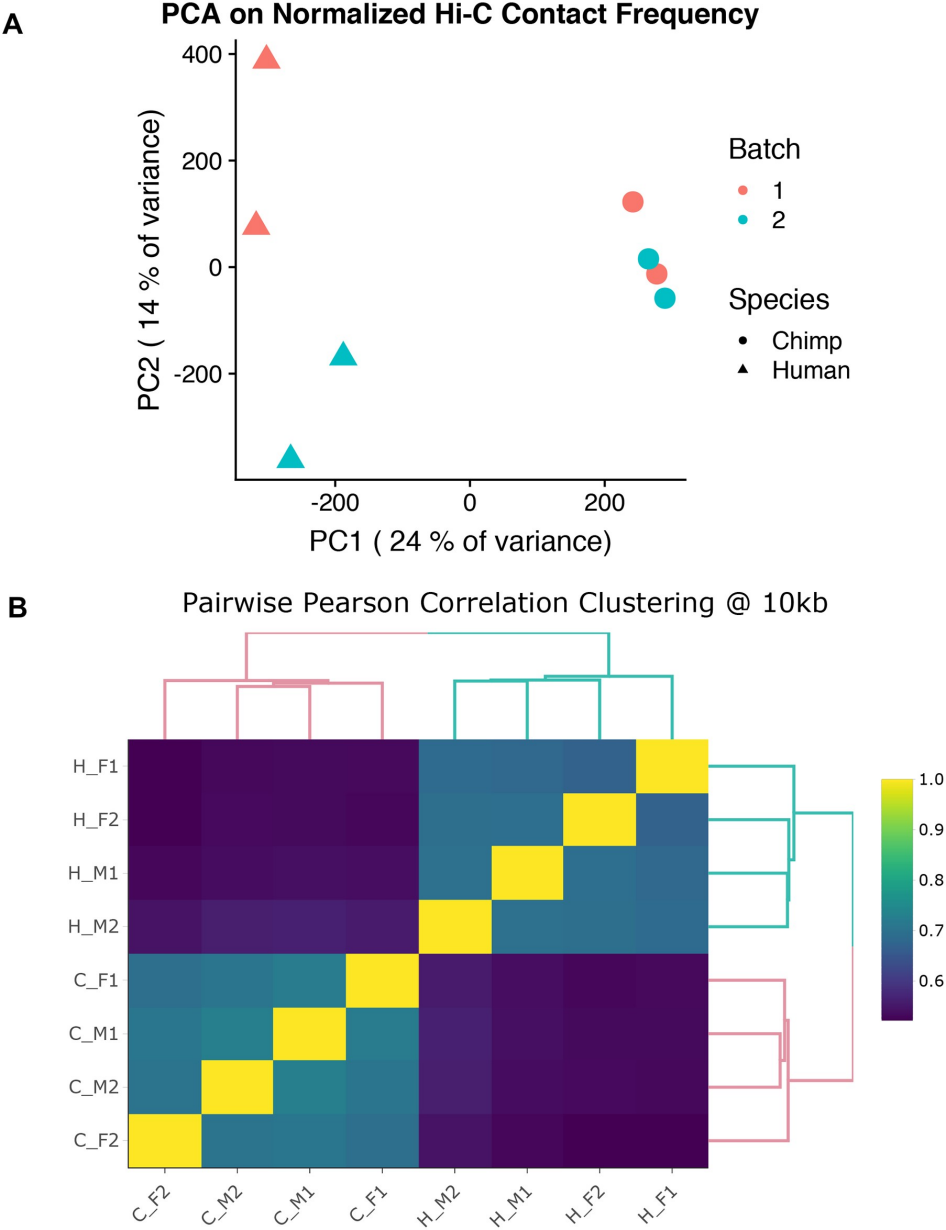
General patterns in Hi-C data. (A) Principal components analysis (PCA) of HOMER-normalized interaction frequencies for the union of all contacts in humans (triangles) and chimpanzees (circles). PC1 is highly correlated with species (r = 0.98; P < 10–5). (B) Unsupervised hierarchical clustering of the pairwise correlations (Pearson’s r2) of HOMER-normalized interaction frequencies at 10 kb resolution. The first letter in the labels demarcates the species (H for human and C for chimpanzee), and the following symbols indicate sex (male, M or female, F) and batch (1 or 2).

We used limma [63] to perform pairwise cyclic loess normalization and minimize the effects of technical variables on our data (S8 Fig). Following normalization, principal components analysis (PCA) and unsupervised hierarchical clustering of the Hi-C data revealed that, as expected, samples cluster by species (Fig 1).

To identify inter-species differences in contact frequencies, we analyzed the data using a linear model with fixed effects for species, sex, and processing batch (see Methods). At an FDR of 5%, we classified 13,572 contacts (about 4%) as having differential normalized contact frequency between humans and chimpanzees. Analysis of the orthologous regions anchoring these contacts suggested that approximately 4,000 of these differences might be explained by large inter-species differences in distance between mates of a contact pair (because read count is correlated with distance between the mates; see Methods and S9 Fig). We thus conservatively excluded locus pairs whose distance varied by more than 20 kb across species. Ultimately, we classified with confidence 9,661 Hi-C contacts (of 292,070; about 3.3%) with a significant difference in normalized contact frequency between the two species. We refer to these contacts as inter-species differentially contacting (DC) regions (S10 Table). Our observations thus suggest that lower-order contacts are generally conserved between humans and chimpanzees. That said, if we assume that all of the contacts we filtered out (either due to lack of orthology or because the distance between the anchor regions differed across species) are in fact DC, divergence in contact frequency would have been observed for 16% of the Hi-C contacts (assuming similar properties to the current data set). However, we find it more likely that a large subset of the contacts we excluded are not truly DC, but, rather, not comparable between the species due to differences in genome assembly quality.

**Fig 2:**
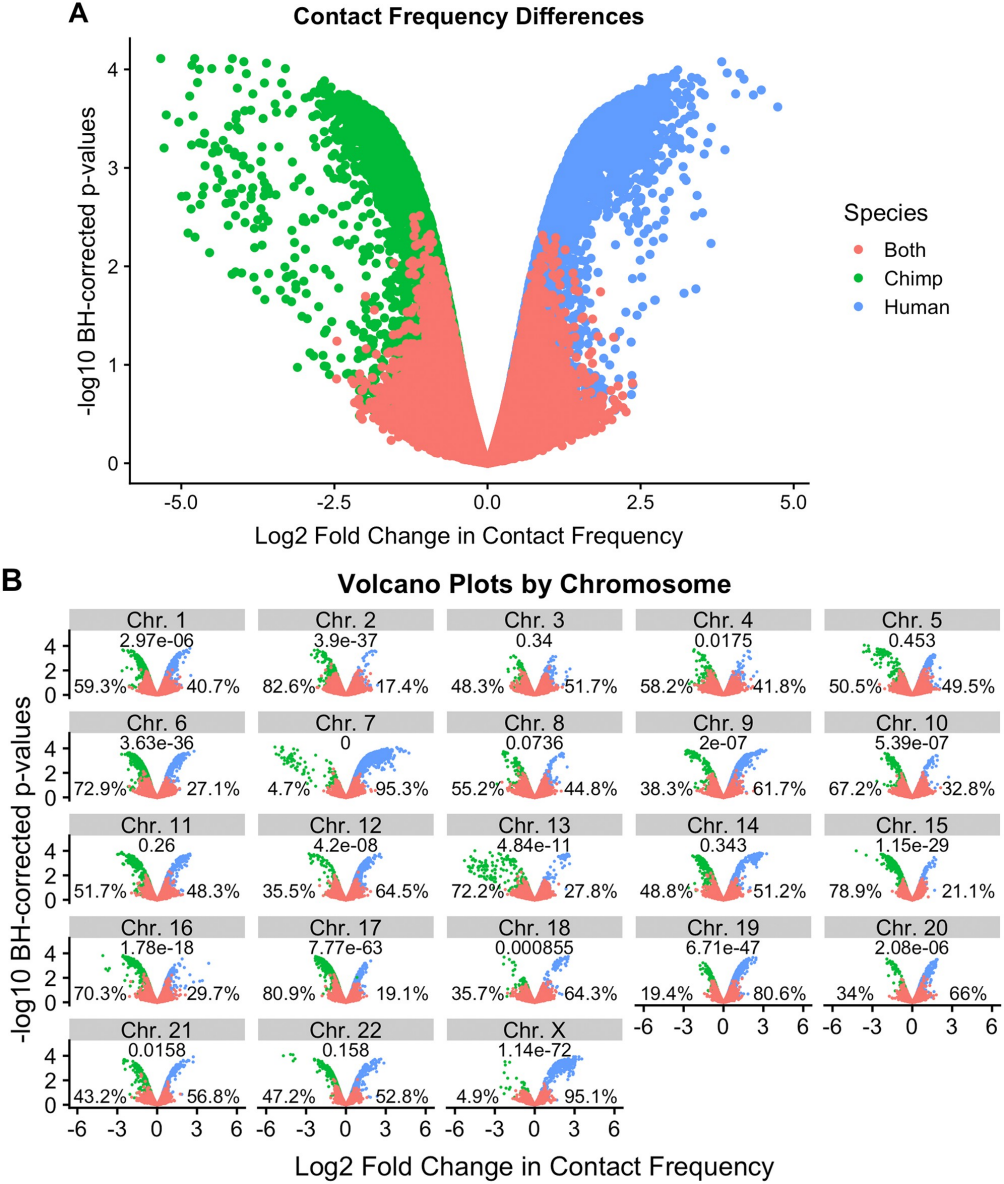
Linear modeling reveals large-scale chromosomal differences in contact frequency. (A) Volcano plot of log2 fold change in contact frequency between humans and chimpanzees (x-axis) against Benjamini-Hochberg FDR (y-axis), after filtering non-orthologus regions (results for unfiltered data are plotted in S9 Fig). Data are colored by the species in which the contact was originally identified as significant. (B) Per-chromosome volcano plot using the same legend as in A. P-values provided for a binomial test of the null that inter-species differences in contact frequencies are evenly distributed. The percentage of contacts with significant higher frequency in each species is noted.

**Fig 3:**
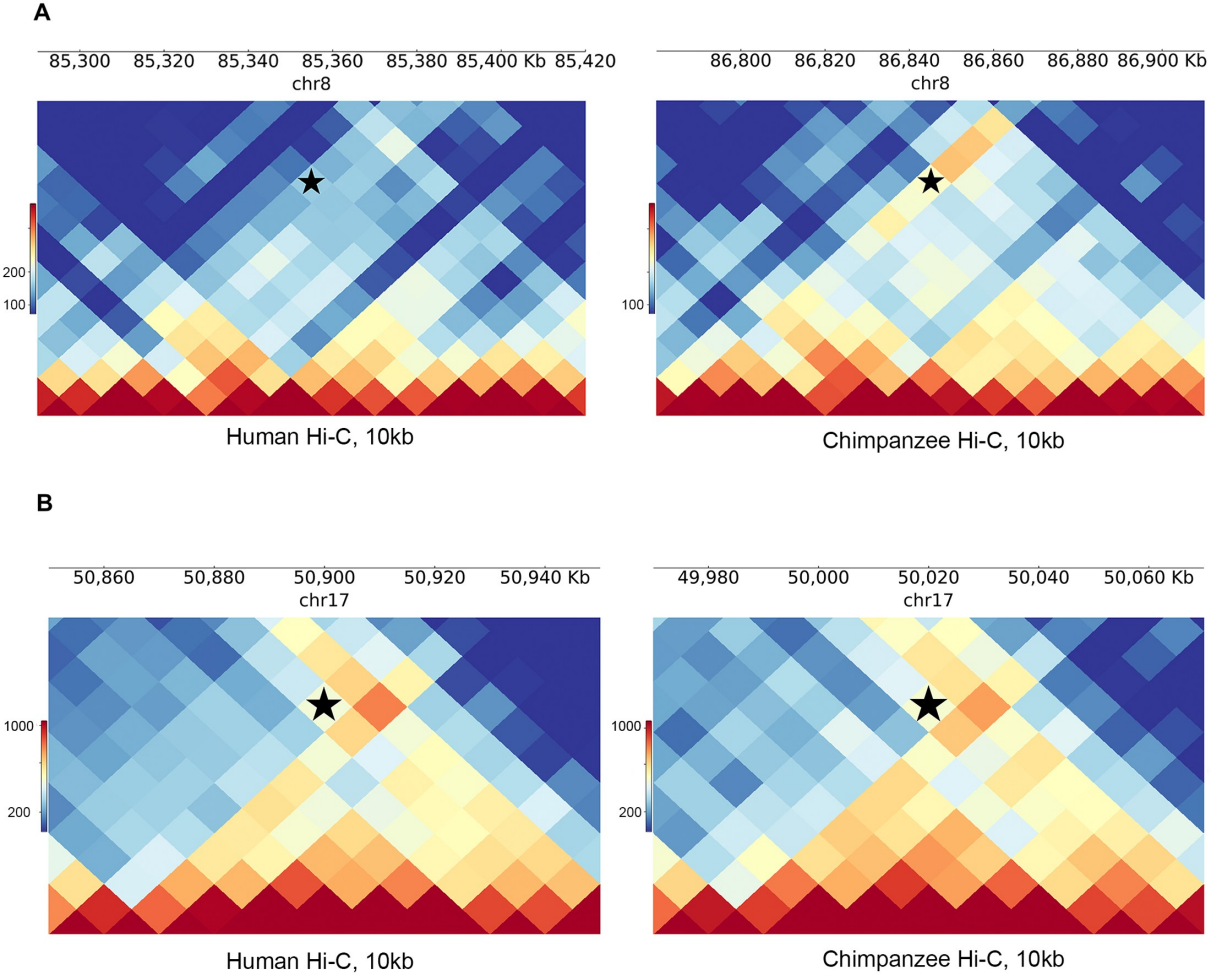
Examples of DC and non-DC Interactions. (A) PyGenomeTracks plots [84] of a chromosome 8 interaction between bins 130kb away for human (left panel) and chimpanzee (right). The bin pair tested is indicated by a black star, and was found to be DC between species. (B) Same as A, but for a conserved (non-DC) interaction on chromosome 17 separated by 100kb.

Across all DC regions, 55% exhibited a higher contact frequency in chimpanzees, while 45% showed a higher frequency in humans (Fig 2A, see Fig 3 and S10 Fig for examples). We observed that some chromosomes were associated with greater asymmetry in inter-species contact frequencies than others (Fig 2B). Greater asymmetry seems to be present more often in chromosomes with large inter-species rearrangements. Specifically, in our data, 8 of the 9 chromosomes with known large-scale pericentric inversions between the species (1, 4, 5, 9, 12, 15, 16, 17, and 18; [53, 54, 64–67]) show particularly strong asymmetry in inter-species contact frequencies. We also observed asymmetry in inter-species contact frequencies in human chromosome 2, a fusion of the ancestral chromosomes giving rise to chimpanzee chromosomes 2A and 2B [66], as well as in chromosome 7, which has the highest number of un-localized sequences of any chromosome in the panTro5 genome.

**Fig 4:**
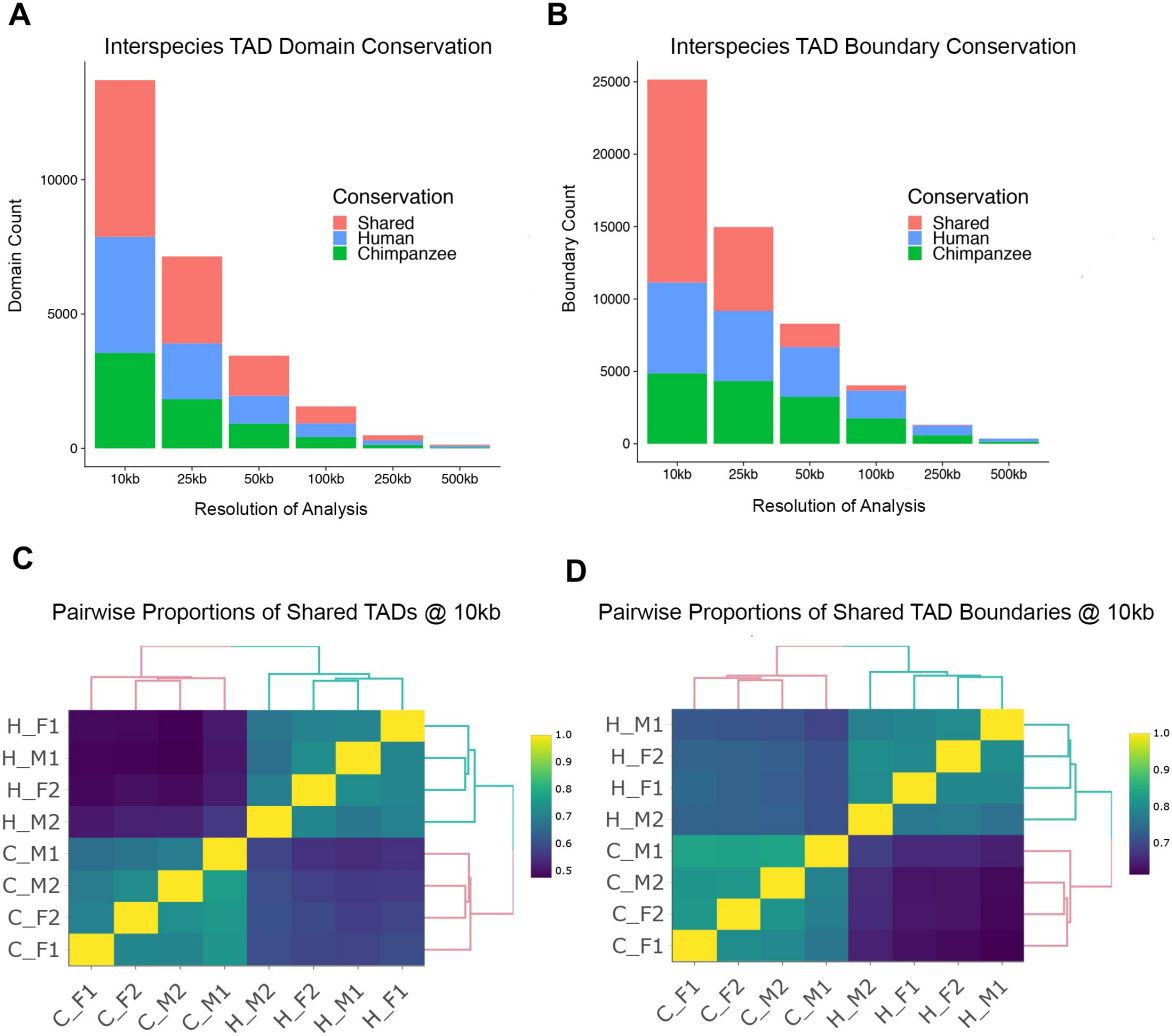
Higher-order chromosomal structure in humans and chimpanzees. (A) Across different resolutions (x-axis), we plotted the number of shared and species-specific domains (y-axis) identified with Arrowhead [62] using the consensus map from each species (alternative approaches plotted in S12–S14 Figs). (B) Same as A, but for TAD boundaries instead of the domains themselves. Boundaries were defined as 15kb flanking regions at the edges of inferred Arrowhead domains. (C) Unsupervised hierarchical clustering of pairwise comparison of TADs across all individuals. These proportions were obtained using Arrowhead TAD inferences on each individual at 10kb resolution. Proportions indicated by color scale on right. Similar plots using analysis at different resolution are available in S12 and S14 Figs. (D) Similar to C, but for TAD boundaries instead of the domains themselves.

Next, we turned our attention to higher-order chromosomal structures by characterizing TADs in each species. Previous studies indicate that the human and chimpanzee genomes share a high degree of synteny [53–58], a property we confirmed by tiling each genome into various bin sizes and using a reciprocal best hits liftOver method to identify syntenic regions (see Methods and S11 Fig). To infer steady-state TAD structures, we pooled reads across all individuals within each species to create “high-density consensus” Hi-C maps for humans and chimpanzees [62]. We used the Arrowhead algorithm at 10 kb resolution [62] to independently infer 11,298 TADs in humans and 10,505 TADs in chimpanzees (see Methods). We then used liftOver to identify orthologous genomic regions that corresponded to these TADs, and removed 10% of domains for which orthology could not be identified (S11 and S12 Tables list the TADs identified in each species; S13 Table lists the orthologous locations of the combined TADs). Once orthology has been established, for each TAD, we considered the domain conserved in humans and chimpanzees when 90% of the TAD interval overlapped reciprocally between species (see Methods). Using this approach, we found that only ~43% of TADs discovered in humans and chimpanzees are shared (Fig 4A).

The observation that TADs are generally not as conserved as practically all other regulatory phenotypes studied in humans and chimpanzees was unexpected. We thus thoroughly tested the robustness of this inference. To do so, we performed a large number of alternative analyses. We analyzed the data at different resolutions (from 10 kb to 500 kb—each time repeating the reciprocal liftOver analysis). We analyzed the data by considering, instead of pooled data, TADs identified independently in a single and in up to 4 individuals within each species (Fig 4C and S12 Fig), and we did this across the different resolutions. We analyzed the data by classifying conservation based on the approach of Rao et al. [45] instead of relying on an overlap of 90% of the domain; we analyzed the pooled data using panTro6 as a reference genome rather than the panTro5 assembly (S13 Fig). We analyzed the data by focusing on boundaries instead of the entire domains (Fig 4B and 4D); we used multiple alternative definitions of boundaries, and repeated this analysis across all resolutions and with boundaries identified in different numbers of individuals within species (S12 Fig). Finally, we identified TADs using an alternative algorithm, TopDom [68], and repeated all of the alternative analyses mentioned above using this algorithm (S14 Fig).

**Fig 5:**
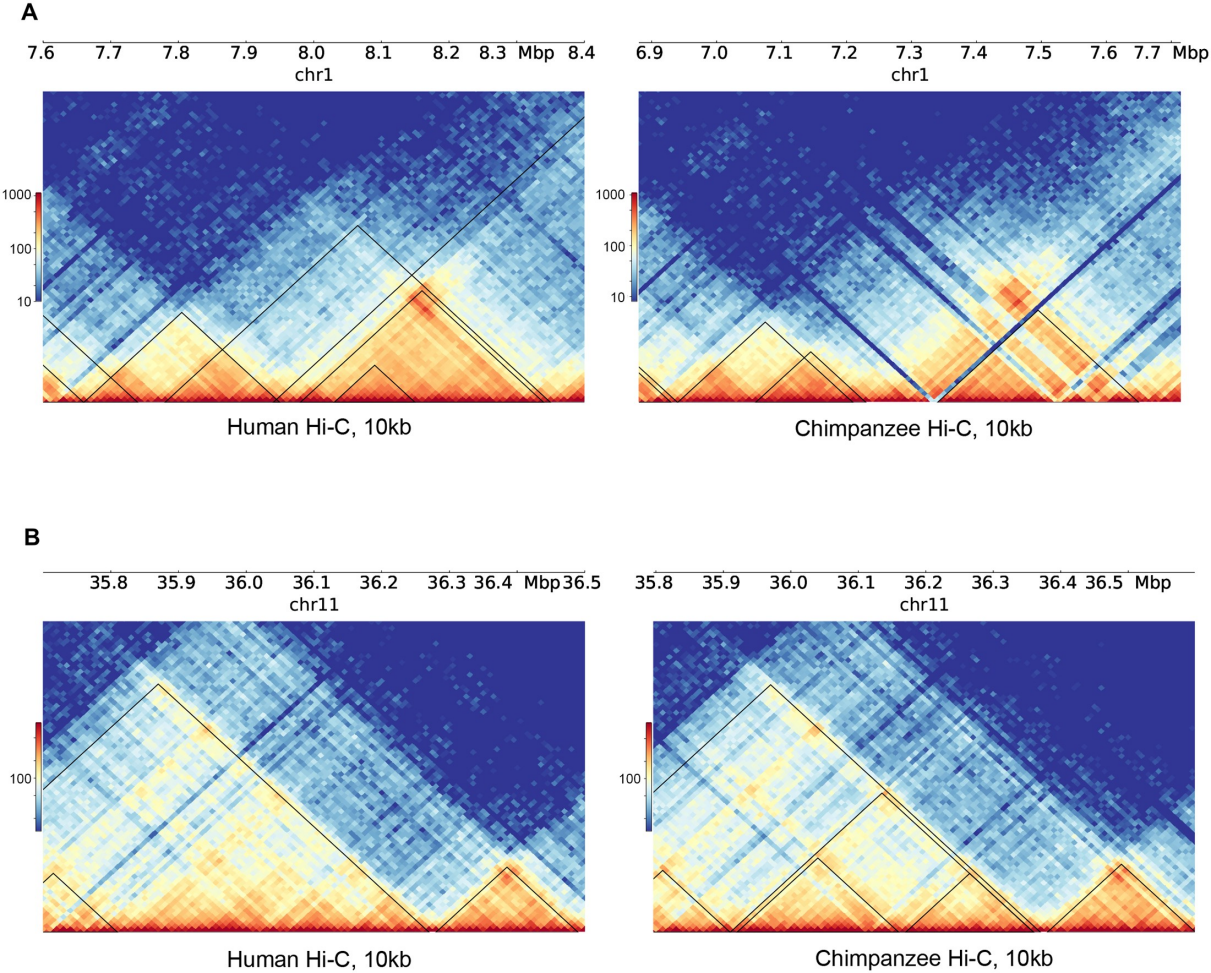
Examples of conserved and divergent TADs. (A) A region on chromosome 1 with examples of both conserved and divergent Arrowhead [62] TAD inferences (black lines). Both the larger TADs seen in the chimpanzee map (right) appear to be conserved in the human map (left), whereas several of the TADs inferred in the human map are noticeably absent from the chimpanzee map. (B) A region on chromosome 11, once again showing examples of conserved and divergent Arrowhead TAD inferences (black lines). All the TADs seen in the human map (left) appear conserved in the chimpanzee map (right), whereas three smaller TADs inferred in the chimpanzee map are not found in the human map, suggesting divergence.

The results of many of these alternative analyses are reported in the supplement (S10 and S12–S14 Figs). All of the alternative analyses produced consistent results and an inference that TADs and TAD boundaries are much less conserved between humans and chimpanzees than any other regulatory phenotype studied to date [69–74]. The Arrowhead analysis of TADs that are independently identified in four individuals within either species, at 10 kb resolution, where conservation is classified based on the less stringent approach of Rao et al. [45], resulted in the highest estimate of conservation, with 78% of domains and 83% of boundaries shared between the species (S12B and S12D Fig). The restriction to TADs or boundaries identified in all 4 individuals of either species results in far fewer features that can be examined (S12 and S14 Figs), yet even in this analysis conservation of domains and boundaries is modest (see Fig 5 and S10 Fig for examples).

### The relationship between inter-species differences in contacts and gene expression

We previously collected RNA sequencing data from the same human and chimpanzee iPSC lines [75]. We jointly analyzed the Hi-C and RNA-sequencing data to learn how often inter-species differences in 3D genomic contact frequencies are associated with inter-species differences in gene expression. We first identified 7,764 orthologous genes for which we have expression and Hi-C data anchored at a region that overlaps the gene’s transcription start site (TSS; see Methods). A single genomic region that overlaps a TSS can have multiple contacts to other genomic regions. For the purpose of our analysis, we conservatively considered only the contact that shows the highest inter-species divergence for each gene.

**Fig 6:**
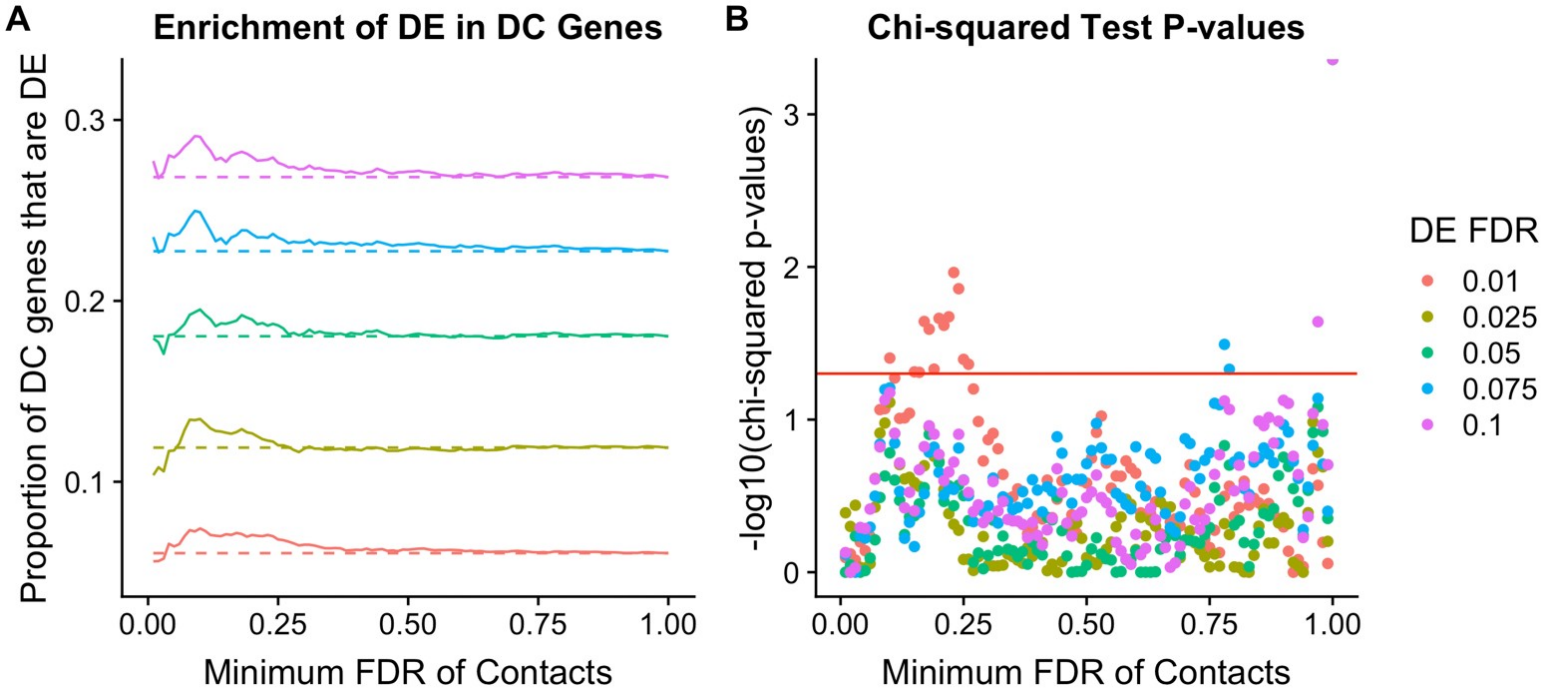
Differentially contacting Hi-C loci show enrichment for differentially expressed genes. (A) Enrichment of inter-species differentially expressed (DE) genes with corresponding differences in Hi-C contact frequencies (DC) between the species. The proportion of DC genes that are significantly DE (y-axis) is shown across a range of DC FDRs (x-axis). Colors indicate different DE FDR thresholds, and dashed lines indicate the proportion of DE genes expected by chance alone. (B) P values of Chi-squared tests of the null that there is no difference in proportion of DE genes among DC genes (y-axis), shown for a range of DC FDRs (x-axis). In both panels, DC regions were chosen to have the minimum FDR supporting inter-species difference in contact frequency. We plotted results using the weighted p-value combination instead of the minimum FDR in S20 Fig.

We did not observe a correlation between gene expression and contact frequency when we considered data from all 7,764 genes. However, when we focused on the 1,401 genes classified as differentially expressed (DE) between humans and chimpanzees (at FDR ≤ 0.05), we observed an excess of both positive and negative correlations between inter-species differences in gene expression and inter-species differences in Hi-C contacts (S15 Fig). Indeed, genes whose TSS is associated with inter-species DC are more likely to be DE between species (χ^2^ test; *P* = 0.01; Fig 6A and 6B). The association between Hi-C contacts and gene expression divergence was somewhat stronger if instead of focusing on the contact with the highest divergence, we obtained a summary *P*-value [76] for testing the null hypothesis that there are no differences between the species in any of the contacts associated with the TSS for a given gene (*P* = 0.001; S20C and S20D Fig).

A combined analysis of functional genomic data does not allow us to infer a direct causal relationship between chromatin contacts and gene expression patterns. Nevertheless, independent evidence strongly suggests that changes in 3D genomic structure can affect interactions between regulatory elements and promoters [45, 77–80], which may ultimately drive differences in gene expression levels [33, 77–82]. We thus sought to quantitatively estimate the extent to which inter-species DC might explain gene expression differences between the species in our data. To do so, we estimated and compared the effect of species on expression before and after accounting for the corresponding contact frequencies (see Methods; [83]).

Specifically, we performed a mediation analysis using linear models to assess the effect of contact on expression divergence (95% confidence interval based on the Monte Carlo test of significance; see Methods). For approximately 8% of DE genes (116/1401) we were able to reject the null hypothesis that the indirect effect is zero (S16 Fig). Taken together, these data suggest that a subset of inter-species differences in gene expression levels can be explained by divergence in Hi-C contacts.

### The chromatin and epigenetic context of inter-species differences in 3D genome structure

Finally, we reasoned that species-specific contacts (i.e. significant DC regions) would be more likely to involve active, functional regulatory elements. This seems intuitive if one assumes most genomic contacts are functionally relevant, and not simply the result of pure noise. To test this hypothesis, we assessed the overlap between our Hi-C data and publicly available chromHMM annotations based on histone modification data from human embryonic stem cells [18]. We assigned each Hi-C locus to an epigenetic state based on its maximum weighted base pair overlap with 15-state chromHMM annotations (see Methods and S17 Fig). Our approach to classify Hi-C regions with a functional assignment based on majority sequence overlap is arbitrary, but our conclusions are robust with respect to alternative approaches to analyze the Hi-C data (S4 and S17 Figs).

**Fig 7:**
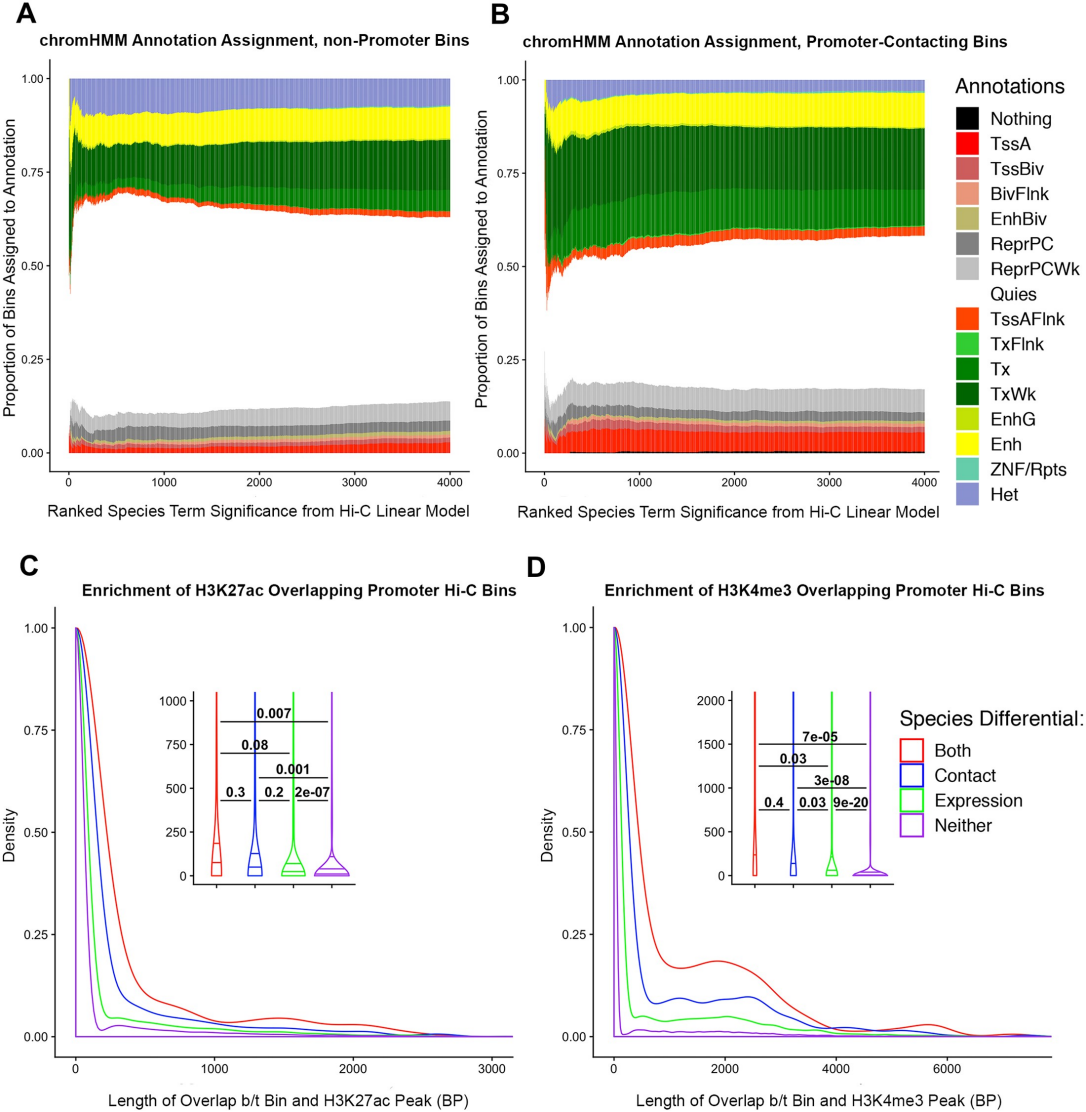
Overlap of epigenetic signatures and Hi-C contacts. (A) Hi-C loci that do not make contact with promoters are ranked in order of decreasing DC FDR (x-axis). The y-axis shows cumulative proportion of chromHMM annotation assignments for all Hi-C loci at the given FDR or lower. (TssA-Active TSS, TSSBiv-Bivalent/Poised TSS, BivFlnk-Flanking Bivalent TSS/Enh, EnhBiv-Bivalent Enhancer, ReprPC-Repressed PolyComb, ReprPCWk-Weak Repressed PolyComb, Quies-Quiescent/Low, TssAFlnk-Flanking Active TSS, TxFlnk-Transcription at gene 5’ and 3’, Tx-Strong transcription, TxWk-Weak transcription, EnhG-Genic Enhancers, Enh-Enhancers, ZNF/Rpts-ZNF genes and repeats, Het-Heterochromatin). (B) Same as A, but only considering Hi-C loci making contact with promoter bins. Results of separating promoter-contacting bins between DE and non-DE genes can be seen in S19 Fig. (C) Density plot of the base pair overlap between different classes of Hi-C contact loci and H3K27ac. Histone mark data were obtained from ENCODE in experiments carried out in human iPSCs. We grouped contacts into 4 classes, indicated by color: those that show differential contact between species, those that show differential expression between species, those that show both, and those that show neither. We used pairwise t-tests to compare differences in the mean overlap among the four classes of Hi-C loci. (D) Same as C, but performed on H3K4me3 data obtained from ENCODE, collected in hESCs. Results with other histone marks can be seen in S18 Fig.

We found marked differences in the chromHMM annotations between genomic regions that are inferred to physically contact a promoter and those that do not contact a promoter (Fig 7A and 7B). For example, genomic regions in physical contact with a promoter are enriched with genic enhancer annotations (χ^2^ test; *P* = 0.0002, S14 Table), as might be expected. Perhaps more novel is the observation that inter-species DC regions were also enriched with genic enhancers, in contrast to regions that did not differ in contact frequency between the two species (*P* = 0.04, S14 Table). We note that this latter observation is not robust with respect to different annotations of enhancers, and we do not find this association if we simply combine all regions annotated as ‘enhancers’ in the data set.

We repeated the enrichment analysis of Hi-C regions using existing human iPSC histone mark data, including H3K27ac, H3K4me1, and H3K4me3, and h1-hESC histone mark data, including H3K27me3 and DNase I hypersensitivity sites (DHS [18]). As expected, Hi-C regions in contact with a promoter showed greater overlap with DHS peaks than Hi-C regions that did not contact a promoter (t-test, *P* < 2.2 * 10^−16^; S18A Fig). When we focused on contacts involving a promoter, we found that inter-species DCs that are also associated with DE genes showed the largest overlap with DHS peaks, followed by DE genes that were not associated with DC regions (*P* = 0.01). Regions that were not associated with either DC or DE showed the least amount of overlap with DHS (*P* = 0.0006; S18B Fig).

Remarkably, apart from the heterochromatic, repressive marker H3K27me3 (where the sign of the effect was the same, but the enrichment was not significant), Hi-C regions that are DC and are also associated with DE genes are more likely to overlap all other histone marks in our data set compared with Hi-C regions that are not DC and are not associated with a DE gene (all enrichment *P* < 0.03; Fig 7C and 7D, S18C and S18D Fig). In other words, inter-species DCs associated with DE genes are more likely to occur in genomic regions that are marked by histone modification, and are thus likely to have a regulatory function.

## Discussion

In general, we observed lower-order, pairwise chromatin contacts in iPSCs to be conserved between humans and chimpanzees. We believe that this observation is intuitive, though we acknowledge that with only four individuals from each species, and given the challenges in identifying orthologous regions, we are likely to somewhat underestimate the degree of divergence in pairwise chromatin contacts.

In contrast to the conservation of lower order pairwise contacts, we did not find higher-order chromatin structures, such as TADs and TAD boundaries, to be generally conserved between human and chimpanzee iPSCs. Because this observation seems to contradict previous reports suggesting that TADs are strongly conserved across species [45, 46], we performed a large number of alternative analyses to demonstrate the robustness of our inference. Even in our most lenient analysis, we observed that only 78% of domains are shared between humans and chimpanzee—a much lower conservation than observed for any other regulatory phenotypes between these two species (when similar sample sizes are considered; [69–73]).

While all of the alternative analyses supported our inference, these analyses also demonstrated the known difficulty of robustly inferring TADs and TAD boundaries based on Hi-C data alone [84, 85]. Indeed, the algorithms used to infer TADs and TAD boundaries themselves are not very robust, as has been discussed previously [85, 86]. Given our observations and the difficulty obtaining robust definitions of TADs and TAD boundaries, we carefully examined the previous evidence for high conservation of TADs between species.

The conclusion of our literature analysis is that the evidence for strong domain conservation is weak, and thus that our inference does not actually contradict previous data. A few of the studies typically cited as providing evidence for strong conservation of TADs across species did not actually perform a genome-wide assessment TADs, but inferred conservation based on a few examples [44, 87, 88]. Rudan *et al*. [44], for instance, reported functional conservation of TAD boundaries in liver cells from rhesus macaque, dog, rabbit, and mouse, but did not report the number or proportion of conserved regions they observed. Instead, they presented correlations of ~0.5 between contact frequencies across these species in subsets of contacts binned by the distance between mates, without further considering TADs or boundaries.

In contrast to studies that focused on specific examples, Dixon *et al*. [46], who originally described megabase-sized TADs at 40 kb resolution, reported that TAD boundaries were conserved in human and mouse embryonic stem cells. At a greater sequencing depth and finer resolution (1 kb), Rao *et al*. [45] observed TADs with a median size of 185 kb, and similar to Dixon *et al*., concluded that the domains were conserved in human and mouse B-lymphoblasts. However, the evidence for conservation in both studies is not strong. First, the actual conservation reported, though described as high, seems in fact to be modest: Dixon *et al*. reported that 54% of human boundaries are shared with mouse (76% if the comparison is reversed), and Rao *et al*. reported that 45% of mouse domains are shared with human. Second, and more importantly, in both studies, conservation estimates were made unilaterally, by considering the proportion of TADs identified in the species for which they had less data that are also identified in the species for which they had more data. This approach results in an overestimate of sharing of domains because only the very strong TADs can be identified in the species with less data, and these are more likely to be shared across species. Indeed, if we perform a similar analysis using our own data (assessing sharing of the top 10% of TADs identified in one species), we observe a much higher conservation (85%). Conversely, if we use the data from Rao *et al*. to estimate reciprocal TAD sharing across species, conservation is even lower than originally reported, at ~30% instead of 45%.

Thus, based on our analysis of the literature, we believe that the common notion that TADs are highly conserved in their placement across species is not well supported. Indeed, recent evidence from yeast [89], different *Drosophila* tissues [90], and plant species [52] suggests that TADs and TAD-like domains may not be particularly conserved, which raises questions about the stability of these higher-order structures and the significance of their role in the evolution of gene regulation across different lineages. However, the extent (or lack) of inter-species TAD conservation is difficult to falsify with existing data, partially because there is no standard method for identifying TADs, nor for comparing them across species [85, 86]. The ability to reliably identify TADs also depends on the quality of the genome assemblies used, the approach for inferring synteny, sequencing depth and coverage, and various other parameters. We acknowledge that our estimates of inter-species differences in TADs may be somewhat inflated due to incomplete power to detect TAD structures in each genome. Unfortunately, the outputs of the available algorithms do not allow us to directly address this potential caveat in the same way we addressed incomplete power in the comparative analysis of lower-order interactions.

More generally, as many studies indicated [85, 91, 92], including ours, it is difficult to reconcile the visual examination of contact maps with TADs inferred based on algorithms. In our case (Fig 5 and S10 Fig), we found many examples where visual inspection naively suggests high conservation, but the algorithms do not indicate sharing of domains or boundaries. This is not surprising; a previous comprehensive analysis of numerous TAD algorithm inferences found very little concordance when compared to manual visual annotations of TADs [85]. Obviously, comparing all TAD inferences based on manual visual assessment is not feasible. Yet, the lack of stability of TAD algorithms means that it is possible that a better computational analysis will emerge and will indicate that domains or boundaries are indeed conserved. Currently, however, neither our own nor previously published data provides support for strong conservation of these structures.

### Contribution of variation in 3D genome structure to expression divergence

We considered our Hi-C data along with gene expression data previously collected from the same cell lines [75] and assessed the extent to which inter-species variation in 3D genome contacts could potentially explain gene expression divergence between species. Previous studies have observed that spatial co-expression of genes is associated with chromatin interaction profiles [26, 30, 31, 41, 93]. A number of studies have focused on differentially expressed genes following a treatment or perturbation and observed that such genes are often associated with corresponding differences in nearby chromatin contacts [77, 78]. Consistent with these reports, we found an enrichment of inter-species differences in pairwise chromatin contacts that involve promoters of differentially expressed genes between the species. Our observations are robust with respect to a range of data processing decisions and the statistical cutoffs we used. Under the common assumption that changes in chromatin contacts are more likely to explain differences in gene expression than vice versa, our results support the notion that species-specific 3D genomic contacts play an important role in the evolution of gene regulation.

Our observation that inter-species differences in pairwise genomic contacts are associated with regulatory evolution more than differences in large scale TAD boundaries is also consistent with previous reports. For example, Rao *et al*. [33] found that the degradation of cohesin, one of the proteins involved in maintaining TAD boundaries and large-scale loops, is associated with only modest effects on gene expression. In contrast, a number of other studies found strong correlations between differences in fine-scale genomic contacts and differences in the expression of nearby genes [45, 79].

Previous studies have identified a wide variety of regulatory phenotypes that contribute to inter-primate differences in gene expression levels [10, 11, 13, 69, 94–96]; 3D genome conformation is only one of the putative upstream factors in the evolution of gene regulation. Our results argue for a model whereby inter-species differences in pairwise contact frequencies are among the main drivers of expression divergence between humans and chimpanzees. Given the low 10-kb resolution of our Hi-C data, it is likely that we have underestimated the contribution of inter-species variation in 3D genome structure to gene expression divergence between species. Future comparative Hi-C studies that sequence deeply enough to obtain higher, sub-kilobase resolutions, will allow researchers to resolve variation in contact frequency at even smaller scales, augmenting predictive power.

### Functional annotations

Finally, we considered our data in the context of functional chromatin annotations available for the human genome. Previous studies have shown that 3D contact maps produced by Hi-C can be accurately recapitulated by epigenetic marks [97, 98]. Other reports have found enrichments for various chromatin accessibility and histone marks among interactions inferred from chromosome conformation capture data [99, 100].

Our results corroborate and expand upon these findings. The differences we observed in chromHMM state assignments in our comparisons (namely, more active and less repressive states in promoter-involved contacts and contacts overlapping differentially expressed genes), provide additional support for the functional relevance of our inferences. We acknowledge that these differences could potentially be more pronounced with higher-resolution Hi-C data and with chromHMM inferences made from ChIP-seq experiments in the same cell lines. While our study design does not allow us to directly infer causality between chromatin interactions and gene expression, the functional enrichments we observed for different epigenetic marks suggest that 3D genome conformation may be one of the upstream elements in the chain of events driving the evolution of gene expression. Although this notion is intuitive to us and is consistent with our data, it is still possible that differences in epigenetic marks are the true drivers of divergence in gene expression levels and/or chromatin contacts between humans and chimpanzees.

Future studies integrating similar data types could explore these possibilities by examining epigenetic marks across species (only human data were available to us), which would enable researchers to polarize the regulatory differences in orthologous sequences between humans and chimpanzees. This would also allow for a sharper definition of the functional classes of inter-species differences in lower-order chromatin contacts.

## Materials and methods

### Ethics statement

We collected human fibroblasts with written informed consent obtained from all human participants under University of Chicago IRB protocol 11–0524. We obtained fibroblasts from chimpanzees from the Yerkes Primate Research Center of Emory University under protocol 006–12, in full compliance with IACUC protocols [59]. All experimental methods are in accordance with the Helsinki Declaration.

### Induced pluripotent stem cells (iPSCs)

As described previously, the Gilad lab has derived panels of both human and chimpanzee iPSCs via episomal reprogramming [59]. To ensure their quality, we validated iPSCs from both species as pluripotent at high passages (>10). Quality control checks included an embryoid body assay confirming their ability to differentiate into all three germ layers, qPCR of endogenous transcription factors associated with pluripotency, PCR to confirm the absence of exogenous pluripotency genes (both from residual episomal plasmid or genomic integration), and PluriTest [101], a bioinformatics classifier that assesses pluripotency based on gene expression data [59]. In the current study, we grew all cell lines in the same incubator in two passage-matched batches, which were also balanced across species and sex, in order to avoid batch effects in our data.

### In-situ Hi-C library preparation and sequencing

We performed *in situ* Hi-C with the restriction enzyme MboI, as previously described [45] on the iPSCs from both species. We grew cells in feeder-free conditions [102] to approximately 80% confluence before adding formaldehyde to crosslink the proteins mediating DNA-DNA contacts. We flash-froze pellets of 5 million cells each before beginning the *in situ* Hi-C protocol [45]. We used MboI to cut the DNA at each of its 4-bp recognition sites (GATC) throughout the genome. Ligation of proximal fragments with T4 DNA ligase yielded chimeric DNA molecules representing two distinct loci. Libraries were created in two balanced batches identical to the cell growth batches and sequenced (100bp paired-end) on an Illumina Hi-Seq 4000 at the University of Chicago Genomics Core Facility. To avoid batch effects resulting from differences in flow cells, libraries were sequenced across three lanes, each on separate flow cells balanced for species.

### Hi-C read mapping, filtering, and normalization

We preprocessed, mapped, and filtered the resulting FastQ sequence files using HiCUP version 0.5.9 [60]. We also used HiCUP to truncate the reads at ligation junctions. Thereafter, we used bowtie2 version 2.2.9 [103] to independently map the two mates of paired-end sequences to either the hg38 or panTro5 genomes, and removed reads with low quality scores (MAPQ < 30). We carried out further HiCUP filtering as previously described based on an *in silico* genome digest in order to remove experimental artifacts [60]. We then used HOMER version 4.9.1, a foundational statistical analysis suite for Hi-C data [61], to tile the genome into a matrix of 10 kb bins and assign reads to their corresponding intersecting bins. We subsequently used HOMER to normalize Hi-C contact bins as previously described [61], accounting for known technical biases in Hi-C data. Finally, we called statistically significant interactions independently in each individual using HOMER, based on a null expectation of read counts falling into bins in a cumulative binomial distribution [61]. We retained interactions with an unadjusted *P* value ≤ 0.01, the default recommendation by HOMER. As other studies have noted [62], a traditional multiple testing correction paradigm is overly conservative for Hi-C data due to the high number of tests, and because the spatial nature of the data means that individual tests are highly correlated (and thus not independent).

### Creation of a union list of orthologous Hi-C contacts across species

In order to ensure that the contact frequencies we compared across species were from representative orthologous sequences in humans and chimpanzees, we used liftOver with a reciprocal best hits method [104, 105] to transfer interaction bin coordinates across both genomes. For each called contact, we used liftOver to independently map the coordinates of the two anchor bins in the other species’ genome, obtaining coordinates in both genomes for all contacts. We then rounded the coordinates to the nearest 10 kb bin, in order to align properly with a Hi-C bin. We required both anchor bins to have orthologous bins in the other species in order to retain a contact for comparison; statistics on the number of called contacts and the number retained after our liftOver procedure are available in S9 Table. In order to assess the extent of contacts lost due to lack of orthology, we also compared the retention of genome-wide 10 kb bins in both genomes with the retention of unique 10 kb bins found within each of our individuals. We found that our Hi-C bins tended to have a higher rate of orthologous mappability across species (S9 Table). For all contacts in this union list, we then extracted the HOMER-normalized interaction frequencies from each individual’s 10 kb Hi-C matrix. Including interactions discovered in fewer than 4 individuals increased the variance in our data (S7 Fig). Therefore, we retained only the Hi-C contacts that were independently discovered by HOMER in at least 4 individuals, for a total of 347,206 interactions. As we describe in the next section, we also later filtered out contacts where the distance between bins showed a difference of > 20 kb across species, retaining 292,070 interactions.

### Linear modeling of Hi-C interaction frequencies

In an effort to quantify inter-species differences in the Hi-C interaction frequency values, we used the following linear model:

$${\text{Y}}_{\text{ij}}=\beta_{0}+\beta_{\text{sp}}{\text{s}}_{\text{i}}+\beta_{\text{sx}}{\text{x}}_{\text{j}}+\beta_{\text{btc}}{\text{b}}_{\text{i}}+\varepsilon_{\text{ij}}$$ (1)

Y_ij_ represents the observed Hi-C interaction frequency of a contact from individual j in species i. β_0_ is the intercept. β_sp_, β_sx_, and β_btc_ are effect sizes for species, sex, and batch, respectively, with their corresponding variables s_i_, x_j_, and b_i_, and an error term ε_ij_. We used the R/Bioconductor package limma [63, 106] to test for inter-species differences in Hi-C interaction frequency. We applied Benjamini-Hochberg multiple testing correction and found 13,572 interaction pairs where the species term is significant at a 5% false discovery rate (FDR).

Initial visualization of the linear modeling results for the species term revealed a stark asymmetry (S9A Fig) suggesting that on a global level, the contacts identified as significant in chimpanzees were much stronger than those identified in humans. This was surprising to us; we reasoned that this asymmetry could be due to a technical factor. For example, liftOver conversion of genome coordinates between species to identify orthologous bins can create differences in both the Hi-C locus size and in the genomic distance between mates of a contact pair (mate-pair distance). We investigated the impact of these two factors on the proportion of contacts classified as differential across species in our data. We discovered that while changes in Hi-C locus size had little effect on the proportion of interspecies DCs, differences in mate-pair distances > 20 kb across species created a noticeable inflation in this proportion at an FDR of 5% (S9B Fig). We believe this makes intuitive sense, as bins that are farther apart will have fewer read counts due to the proximity-based ligation in Hi-C. Thus, a mate-pair distance difference across the genomes could induce what appears to be a differential contact, because the contact inherently has more read support in the species where the mates are closer. However, we note that it is impossible to ascertain the relative biological and/or technical relevance of the differences seen in these contacts. We thus took a conservative approach to minimize false positives and removed contacts with a >20 kb mate-pair distance difference between species from our downstream analyses (S9 Fig), accepting that the number of inter-species differences we observe may be underestimated.

### Identification of orthologous topologically associating domains (TADs) and boundaries

We chose to perform TAD analyses on both individual-level data and on representative species consensus data. For our analysis comparing TAD boundaries on species consensus Hi-C maps, we combined all the preprocessed Juicer files from all our individuals within a species and used the *juicer_mega*.*sh* script [62] to create higher density contact maps for each species. We then ran the Arrowhead algorithm across resolutions to infer TADs, and then we extended the edges of TADs 7.5 kb in each direction to create 15 kb boundaries (accounting for imprecision in boundary inference). We used a reciprocal best hits liftOver strategy [104, 105] to obtain orthologously mappable TADs and boundaries. To confirm high synteny of large-scale linear genomic intervals between the species, we employed this same orthology analysis on genome-wide tilings of the hg38 and panTro5 genome assemblies, with varying window sizes created with bedtools [107] *makewindows* (S11 Fig). In the case of TADs, we then assessed number of domains found in one species that were also found in the other species (conserved domains) with reciprocal bedtools [107] *intersect* −*c −f 0*.*9 -r* calls. These parameters will only define a domain as overlapping if there is a domain in the other species such that each domain shares 90% of their interval with the other. We used the larger of the two estimates of shared TADs across the species as the conserved domain count (to be conservative), and divided this by the sum of the conserved and species-specific domains identified in order to assess conservation. As an alternative analysis, we also employed the method previously described by Rao *et al*. [45]; namely, we called a TAD conserved in one species if it and a TAD from the other species displayed a Euclidean distance less than the smaller of 50 kb or half the given TAD’s size. We analyzed boundary conservation using bedtools *intersect*–*c*, considering any overlap as indication of conservation (i.e. even a single base pair overlap of boundaries meant a boundary was classified as conserved).

To examine individual-level data and to ensure robustness of our results, we separately used both Arrowhead [62] and TopDom [68] (with *window = 20*) across resolutions to call TADs independently in each individual sample. Though we performed essentially the same analyses on both outputs, it should be noted that Arrowhead provides nested TADs only, from which we inferred boundaries as described above, whereas TopDom provides separate domain and boundary inferences. We used a reciprocal best hits liftOver method [104, 105] to obtain a set of orthologous domains and boundaries. We assessed interspecies conservation by performing left outer joins (bedtools *intersect—loj*) of each individual’s domains against all the others, once again requiring 90% reciprocal overlap. We then took the average species-specific and shared domain counts across these individual comparisons to produce a single estimate of conservation (S12 and S14 Figs). The individuals’ pairwise percentages of shared domains were used in hierarchical clustering analysis (Fig 4C, S12 and S14 Figs). We also once again checked the robustness of our results using the conservation calling method from Rao *et al*. [45] described above. In the case of boundaries, we reasoned that, given the nested nature of the TADs, as well as variance between individuals in their exact placement, it would make sense to merge the boundaries (using bedtools *merge*) in order to obtain a list of unique boundary elements. We added a column of individual identifiers to each set of boundaries and then merged all together, thereafter assessing conservation by examining what percentage of boundaries were independently found in both species out of the total set of unique boundaries. We also applied hierarchical clustering analysis to individual pairwise percentages of shared boundaries in this union merged file (Fig 4D, S12 and S14 Figs). Further descriptions of these analyses can be found on our GitHub repository (/data/TADs folder), and 10 kb individual Arrowhead inferences are available in S17–S24 Tables.

### Differential expression analysis

Previously, the Gilad lab generated RNA-seq expression data on the same iPSC lines from this study (GEO accession GSE110471 [75]). We computed reads per kilobase per million mapped reads (RPKM) for every gene, as the orthologous genes are not constrained to be the same length across species. We retained 11,074 genes that had at least half of the individuals (2 observations) in each species with log_2_ RPKM ≥ 0.4. We then used the limma-voom pipeline with precision weights [63, 106] to test for differential expression across species, using a linear model including a species effect and a sex effect. Using this approach, we found 2,086 differentially expressed genes (at 5% FDR).

### Broad integration of Hi-C and gene expression data

We obtained the overlap between our gene expression data and our Hi-C data by applying bedtools *overlap* [107] to the Hi-C loci and the first exon of each gene. Using a curated file of orthologous gene coordinates between humans and chimpanzees [75], we extracted a one-base-pair interval at the beginning of each first exon to use as a proxy for transcription start sites (TSSs).

As described in the main text, the difference in dimensionality between the two datasets presented a challenge. While every gene has only one expression value per individual, a given Hi-C locus can and frequently does make contact with many other loci. When a given gene overlapped a Hi-C locus making multiple contacts, we chose the contact with the smallest species term FDR (i.e. the most species-specific contact) in our DC analysis to represent the interaction frequency for that gene. Accordingly, we interpreted the FDR-adjusted *P* value for the chosen contact as the gene’s differential contact significance. To examine correlations between normalized Hi-C contact frequency and log_2_ RPKM gene expression, we considered the correlation between gene expression values across all 8 individuals with the corresponding interaction frequency values across the same 8 individuals.

### Enrichment of differential expression in differential contacts

We examined the enrichment of differential expression in genes with differential contact (Fig 6A and S20C Fig) across a continuous range of DC FDRs and a discrete range of DE FDRs (1%, 2.5%, 5%, 7.5%, and 10%). We used Pearson’s chi-squared test to quantify significance of the enrichment at each FDR (Fig 6B and S20D Fig). We also examined the reciprocal enrichment; that is, DC enrichment amongst DE genes (S20A and S20B Fig).

### Assessing the quantitative contribution of Hi-C contact frequencies to gene expression levels

We assessed the hypothesis that expression divergence may be mediated by contact frequency using linear models [83]. The intuition behind this approach is that the effect of species (X) on expression (Y) can be partitioned into its indirect effect on expression mediated through contact frequency (M) and its direct effect on expression. Therefore, a significant indirect effect would suggest that expression divergence is causally mediated by contact frequency. To test our mediation hypothesis, we computed the indirect effect of species on expression (X -> M -> Y: causal effect of X on Y through M) by taking the product of the effect of species on contact frequency (α: X-> M) and the effect of contact frequency on expression after controlling for species (β: M -> Y). The indirect effect (α*β) is conceptually equivalent to the difference between the effect of species on expression and the effect of species on expression after controlling for contact frequency, but is more mathematically tractable and commonly used in mediation analyses [108–110]. We obtained α as the species effect size in a simple linear model attempting to predict Hi-C interaction frequency based solely on a species term. We estimated β as the contact frequency effect size in a linear model predicting expression based on both species and contact frequency per gene. To determine statistical significance of the indirect effect, we applied the Monte Carlo test of significance to construct the 95% confidence interval. The primary benefits of the Monte Carlo method are that it requires no distributional assumptions of the data and is robust against type I error in small samples [111–113]. Thus, we choose the Monte Carlo test over Sobel test, the conventional approach to significance testing of mediation, which relies on the data following normal distribution [108, 109].

### Integration with epigenetic annotations

We obtained chromHMM 15-state model peak calls in human iPS-18C cells from ENCODE [18] (S15 Table). We subsequently found the overlap between the human coordinates of our orthologous Hi-C contact loci and the chromHMM peak calls and quantified the extent of base pair overlap between each locus and all overlapping chromHMM peaks. We assigned each individual locus a single chromHMM annotation based on the peak with the highest base pair overlap with that locus. However, the distribution of overlaps of different chromHMM annotation peaks with our Hi-C bins were quite variable in size. To account for this, we normalized each annotation’s overlap length in each locus by multiplying it by the reciprocal of its mean base pair overlap across all our bins (S17 Fig). After removing duplicate Hi-C loci, we then assigned individual loci to chromHMM annotations based on these normalized base pair overlaps. We started with a small set of the top ten most differentially contacting loci (i.e. the ten lowest FDR loci from our Hi-C linear modeling), and tabulated proportions of which annotations were represented amongst them. We then iteratively added the next-lowest FDR contact (i.e. two Hi-C loci at a time) to this tabulation, re-calculating proportions on the new set of contacts. We ran this same cumulative proportions analysis separately on contacts not overlapping promoters, contacts overlapping promoters, contacts overlapping promoters of DE genes, and contacts overlapping promoters of genes that were not DE (Fig 7A and 7B, S19 Fig).

We also obtained data on H3K4me1, H3K4me3, and H3K27ac collected in human iPS-18A cells, and data on H3K27me3 and DNase hypersensitivity sites collected in H1-hESCs, all from ENCODE [18] (S15 Table). We used bedtools *intersect* [107] to find the base pair overlap between each of these different marks and our Hi-C contact loci. We then removed duplicate Hi-C loci from the dataset and used a pairwise t-test to identify significant differences in the overlapping distributions for different sets of Hi-C classes (based on differential contact and differential expression, Fig 7C and 7D).

## Supporting information

S1 Fig Regulatory landscapes cluster by species, Juicer. (A) Principal components analysis (PCA) of Juicer vanilla coverage (VC)-normalized interaction frequencies for the union of all contacts in humans (triangles) and chimpanzees (circles). PC1 is highly correlated with species (*r* = 0.89; *P* = 0.0004). (B) Unsupervised hierarchical clustering of the pairwise correlations (Pearson’s r^2^) of Juicer VC-normalized interaction frequencies at 10 kb resolution. The first letter in the labels demarcates the species (H for human and C for chimpanzee), and the following symbols indicate sex (male, M or female, F) and batch (1 or 2).
(PDF)

S2 Fig Linear modeling reveals large-scale chromosomal differences in contact frequency, Juicer. (A) Volcano plot of log2 fold change in contact frequency between humans and chimpanzees (x-axis) against Benjamini-Hochberg FDR (y-axis), after filtering non-orthologous regions. Data are colored by the species in which the contact was originally identified as significant. (B) Per-chromosome volcano plot using the same legend as in A. P-values provided for a binomial test of the null that inter-species differences in contact frequencies are evenly distributed. The percentage of contacts with significant higher frequency in each species is noted. Of note is that many of the same chromosomal asymmetries in contact strength observed here are in the same chromosomes as those observed in the HOMER-normalized data (Fig 2).
(PDF)

S3 Fig Differentially expressed genes show enrichment for differential Hi-C contacts, Juicer. (A) Enrichment of inter-species differentially expressed (DE) genes with corresponding differences in Hi-C contact frequencies (DC) between the species. The proportion of DC genes that are significantly DE (y-axis) is shown across a range of DC FDRs (x-axis). Colors indicate different DE FDR thresholds, and dashed lines indicate the proportion of DE genes expected by chance alone. (B) P values of Chi-squared tests of the null that there is no difference in proportion of DE genes among DC genes (y-axis), shown for a range of DC FDRs (x-axis). In both panels, DC regions were chosen to have the minimum FDR supporting inter-species difference in contact frequency. (C) Same as A, but this time, a weighted p-value combination technique [76] was used to integrate each Hi-C bin’s DC FDR across all of its contacts. (D) Same as B, but for the weighted p-value combination instead of the minimum FDR contact.
(PDF)

S4 Fig Dynamics of chromHMM state among significant Hi-C contacts, Juicer. (A) Hi-C loci that do not make contact with promoters are ranked in order of decreasing DC FDR (x-axis). The y-axis shows cumulative proportion of chromHMM annotation assignments for all Hi-C loci at the given FDR or lower. (TssA-Active TSS, TSSBiv-Bivalent/Poised TSS, BivFlnk-Flanking Bivalent TSS/Enh, EnhBiv-Bivalent Enhancer, ReprPC-Repressed PolyComb, ReprPCWk-Weak Repressed PolyComb, Quies-Quiescent/Low, TssAFlnk-Flanking Active TSS, TxFlnk-Transcription at gene 5’ and 3’, Tx-Strong transcription, TxWk-Weak transcription, EnhG-Genic Enhancers, Enh-Enhancers, ZNF/Rpts-ZNF genes and repeats, Het-Heterochromatin). (B) Same as A, but only considering Hi-C loci making contact with promoter bins. (C) Same as B, but only considering Hi-C loci making contact with promoters of genes that are not differentially expressed (DE). (D) Same as C, but only considering Hi-C loci making contact with promoters of genes that are differentially expressed (DE).
(PDF)

S5 Fig Overlap of activating and repressive histone marks among Hi-C contacts, Juicer. (A) Density plot of the base pair overlap between different classes of Hi-C contact loci and H3K27ac. Histone mark data were obtained from ENCODE in experiments carried out in human iPSCs. We grouped contacts into 4 classes, indicated by color: those that show differential contact between species, those that show differential expression between species, those that show both, and those that show neither. We used pairwise t-tests to compare differences in the mean overlap among the four classes of Hi-C loci. Unlike in the HOMER-normalized data, we do not observe statistically significant differences in overlaps with H3K27ac between different locus classes. This may reflect the previous observation that the hiccups algorithm for assigning statistical significance of loops in Hi-C data is much more conservative than HOMER’s significance calling method [86]. (B) Same as A, but performed on H3K4me3 data obtained from ENCODE, collected in hESCs. (C) Same as A and B, but performed on H3K4me1 data obtained from ENCODE, collected in human iPSCs. (D) Same as A, B, and C, but performed on H3K27me3 data obtained from ENCODE, collected in human iPSCs.
(PDF)

S6 Fig Gene expression variance is explained by chromatin contacts for 5% of DE genes, Juicer. Plot of the species effect size in DE genes between models before (x-axis) and after (y-axis) conditioning on contact frequency. The Monte Carlo test of significance was used to construct the 95% confidence interval and evaluate the significance of the indirect effect (species’ effect on expression mediated through contact). Amongst DE genes, 5% (15/299) of genes showed a statistically significant effect of Hi-C contacts on expression levels (i.e. their 95% confidence interval does not include zero).
(PDF)

S7 Fig Variance in interaction frequency as a function of the number of individuals in which a significant interaction is independently discovered. (A) Boxplots of variance in contact frequency across all 8 individuals on the y-axis, binned by the number of individuals in which an interaction is independently called significant on the x-axis. (B) Same as A, but zoomed in on the y-axis to visualize finer-scale variation.
(PDF)

S8 Fig Distributions of HOMER-normalized interaction frequencies are remarkably similar across species. (A) Histogram of log2(observed/expected) HOMER-normalized interaction frequencies in all four human samples used in this study, after applying pairwise cyclic loess normalization with limma [63]. (B) Same as A, but in chimpanzees.
(PDF)

S9 Fig Volcano plot asymmetry quality control. (A) Volcano plot of log2 fold change in contact frequency between humans and chimpanzees (x-axis) against Benjamini-Hochberg FDR (y-axis). This plot shows data only filtered for independent discovery in at least 4 individuals. Data are colored by the species in which the contact was originally identified as significant. (B) Scatter plot of sets of Hi-C contacts, with proportion of contacts significant in our linear modeling of interaction frequency shown based on color. Contacts are binned by mate-pair distance differences (y-axis) and bin size differences (x-axis). Circle size is proportional to the size of the set of Hi-C contacts falling into each criteria. Red indicates that the data were filtered out after this step, and blue/purple indicates that the data were retained for further analysis. (C) Volcano plot as in A, but after removing contacts with large mate-pair distance differences across the species.
(PDF)

S10 Fig Further visual examples of DC and non-DC interactions; conserved and divergent TADs. (A) PyGenomeTracks plots [84] of a chromosome 19 interaction between bins 80 kb away for human (left panel) and chimpanzee (right panel). The bin pair tested is indicated by a black star, and was found to be DC between species. (B) Same as A, but for a conserved (non-DC) interaction on chromosome 1 separated by 100kb. (C-H) Examples of contact maps (created with PyGenomeTracks [84]) and Arrowhead-inferred TAD structures (black lines) in humans (left) and chimpanzees (right), across a number of different chromosomes. In most examples, inference based on the algorithm indicates shared and species-specific domains, yet these are difficult to ascertain based on visual inspection, as discussed.
(PDF)

S11 Fig Synteny of large scale linear genomic intervals between human and chimpanzee. (A) Across different window sizes (x-axis) for a genome-wide tiling of hg38, we plotted the number of total and syntenic linear intervals (y-axis), identified using the reciprocal best hits liftOver method [104, 105] we employed throughout the paper. (B) Same as A, but for a genome-wide tiling of panTro5.
(PDF)

S12 Fig Higher-order chromosomal structure in humans and chimpanzees with alternative analysis choices. (A) Across different resolutions (x-axis), we plotted the number of shared and species-specific domains (y-axis) identified with Arrowhead [62] on Juicer VC-normalized Hi-C maps from each individual. We called domain conservation here based on the method of Rao *et al*. [45] (highly similar results were observed with our 90% reciprocal overlap method, described in the text and available in the github repository associated with the paper). Domain count values represent the average interspecies sharing across all individuals, with no filtering for domain robustness (that is, assessing all domains discovered and orthologously mappable). Under this analysis paradigm we observe relatively low sharing across species (~60% at 10kb). (B) Same as A, but this time, only considering TADs that were found across all 4 individuals within either one of the species (fixed TADs). Restricting to this subset increases the percentage of conservation to 78%, although the set of TADs being examined is much smaller. (C) Same as A, but for boundaries instead of domains. Boundaries were defined as 15kb flanking regions at the edges of inferred Arrowhead domains. Because the TADs called by Arrowhead are nested, we merged boundaries here to obtain unique genomic intervals, rather than counting boundaries repeatedly. We then considered boundaries shared between individuals if they had any overlap. (D) Same as B, but for boundaries instead of domains (i.e. considering only boundaries fixed within species). Here, the highest estimate of conservation we obtain is 83% of boundaries conserved across species at 10kb resolution. (E) Unsupervised hierarchical clustering of the pairwise proportions of shared TADs between all individuals in our study at a variety of resolutions, using the Rao *et al*. [45] methodology for calling conservation. The first letter in the labels demarcates the species (H for human and C for chimpanzee), and the following symbols indicate sex (male, M or female, F) and batch (1 or 2). Heatmaps are not necessarily symmetric because different numbers of TADs were discovered in different individuals; rows represent an individual’s shared proportion of TADs (individual total) with each other individual. Highly similar clustering results were observed when using our domain conservation calling paradigm (shown in github repository associated with paper). (F) Same as E, but for boundaries instead of domains.
(PDF)

S13 Fig Higher-order chromosomal structure in humans and chimpanzees with alternative analysis choices and genome builds. (A) Across different resolutions (x-axis), we plotted the number of shared and species-specific domains (y-axis) identified with Arrowhead [62] using the consensus map from each species. To call a domain as conserved here, we required that the Euclidean distance between the domain across species be less than the minimum of 50kb or 50% the length of the TAD, based on the conservation calling method employed by Rao *et al* [45]. Results are highly similar to those seen in Fig 4A. (B) Same as A, but for TAD boundaries instead of the domains themselves. Boundaries were defined as 15 kb flanking regions at the edges of inferred Arrowhead domains. In this case, conservation was called if there was any base pair overlap between boundaries. Unlike in Fig 4B, boundaries were merged before calling conservation, in order to find unique boundary elements. This difference in analysis paradigms could have important consequences with a nested TAD caller such as Arrowhead [62], but results are highly similar to those seen in Fig 4B. (C) Same as A, but this time, performed on “high-density consensus” Hi-C maps that have been mapped to the hg38 and panTro6 genomes (rather than panTro5). Results are highly similar despite the improvement in genome quality build. (D) Same as B, but this time, on the hg38 and panTro6 genome assemblies.
(PDF)

S14 Fig Higher-order chromosomal structure in humans and chimpanzees with alternative algorithms (TopDom). (A) Across different resolutions (x-axis), we plotted the number of shared and species-specific domains (y-axis) identified with TopDom [68] on HOMER-normalized Hi-C maps from each individual. We called domain conservation here based on the method of Rao *et al*. [45] (highly similar results were observed with our 90% reciprocal overlap method, described in the text and available in the github repository associated with the paper). Domain count values represent the average interspecies sharing across all individuals, with no filtering for domain robustness (that is, assessing all domains discovered and orthologously mappable). Under this analysis paradigm we observe relatively low sharing across species (maximum of 30% at 25 kb). (B) Same as A, but this time, only considering TADs that were found across all 4 individuals within either one of the species (fixed TADs). Restricting to this subset increases the maximum percentage of conservation to 42% at 25 kb resolution, although the set of TADs being examined is much smaller. (C) Same as A, but for TopDom [68] boundary inferences instead of domains. We considered boundaries shared between individuals if they had any overlap. (D) Same as B, but for boundaries instead of domains (i.e. considering only boundaries fixed within species). Here, the highest estimate of conservation we obtain is 76% of boundaries conserved across species at 50 kb resolution. (E) Unsupervised hierarchical clustering of the pairwise proportions of shared TADs between all individuals in our study at a variety of resolutions, using the Rao *et al*. [45] methodology for calling conservation. The first letter in the labels demarcates the species (H for human and C for chimpanzee), and the following symbols indicate sex (male, M or female, F) and batch (1 or 2). Heatmaps are not necessarily symmetric because different numbers of TADs were discovered in different individuals; rows represent an individual’s shared proportion of TADs (individual total) with each other individual. Highly similar clustering results were observed when using our domain conservation calling paradigm (shown in github repository associated with paper). (F) Same as E, but for boundaries instead of domains.
(PDF)

S15 Fig Correlations between Hi-C and expression. Density of Pearson correlations between RPKM expression values and log2 HOMER-normalized contact frequencies across all 8 individuals. Solid lines indicate different sets of the observed data and dotted lines represent 10 permutations of the data. The Hi-C contact frequency chosen is that with the minimum FDR from linear modeling of contact frequency on species (see main text). The strong bimodal distribution of correlations between expression and contact suggests many instances where a contact difference between the species can lead to an increase (enhancer) or decrease (suppressor) of expression in the species where the contact is stronger.
(PDF)

S16 Fig Gene expression variance is explained by chromatin contacts for 8% of DE genes. Plot of the species effect size in DE genes between models before (x-axis) and after (y-axis) conditioning on contact frequency. The Monte Carlo test of significance was used to construct the 95% confidence interval and evaluate the significance of the indirect effect (species’ effect on expression mediated through contact). Amongst DE genes, 8% (116/1401) of genes showed a statistically significant effect of Hi-C contacts on expression levels (i.e. their 95% confidence interval does not include zero).
(PDF)

S17 Fig Using a weighting scheme for chromHMM annotations increases the proportion of transcriptional and enhancer-like annotations. (A) Histogram showing the number of Hi-C loci (y-axis) assigned to each chromHMM annotation (x-axis) using maximum base pair overlap to assign each locus to a state. In the legend, “.” denotes that no annotation was found for a given bin. (TssA-Active TSS, TSSBiv-Bivalent/Poised TSS, BivFlnk-Flanking Bivalent TSS/Enh, EnhBiv-Bivalent Enhancer, ReprPC-Repressed PolyComb, ReprPCWk-Weak Repressed PolyComb, Quies-Quiescent/Low, TssAFlnk-Flanking Active TSS, TxFlnk-Transcription at gene 5’ and 3’, Tx-Strong transcription, TxWk-Weak transcription, EnhG-Genic Enhancers, Enh-Enhancers, ZNF/Rpts-ZNF genes and repeats, Het-Heterochromatin). (B) Same as A, only here, we assigned annotations after weighting chromHMM elements’ overlaps with Hi-C loci by the reciprocal of their mean overlap in all our loci. This approach increases the number of 10kb Hi-C bins that are assigned to chromHMM annotations associated with transcriptional and enhancer activity (i.e. TssA, TssBiv, TssAFlnk, EnhG, Enh).
(PDF)

S18 Fig Overlap of epigenetic signatures and Hi-C contacts. (A) Density distribution of the base pair overlap between DHS peaks downloaded from ENCODE and our Hi-C loci. Plot is split between Hi-C loci that contact a promoter and those that do not. Inlay is a violin plot of the same distributions, with lines and numbers indicating pairwise t-tests of the mean, and their corresponding significance levels. (B) Density plot similar to A, but only considering Hi-C loci involved in contact with a promoter, and separating contacts into 4 classes, indicated by color: those that show differential contact between species, those that show differential expression between species, those that show both, and those that show neither. We used pairwise t-tests to compare differences in the mean overlap among the four classes of Hi-C loci. (C) Same as in B, but for the active histone mark H3K4me1. (D) Same as in B and C, but for the repressive histone mark H3K27me3.
(PDF)

S19 Fig Dynamics of chromHMM state among significant Hi-C contacts overlapping DE or non-DE genes. (A) Hi-C loci that make contact with promoters of genes that are not differentially expressed (DE) across species are ranked in order of decreasing DC FDR (x-axis). The y-axis shows cumulative proportion of chromHMM annotation assignments for all Hi-C loci at the given FDR or lower. (TssA-Active TSS, TSSBiv-Bivalent/Poised TSS, BivFlnk-Flanking Bivalent TSS/Enh, EnhBiv-Bivalent Enhancer, ReprPC-Repressed PolyComb, ReprPCWk-Weak Repressed PolyComb, Quies-Quiescent/Low, TssAFlnk-Flanking Active TSS, TxFlnk-Transcription at gene 5’ and 3’, Tx-Strong transcription, TxWk-Weak transcription, EnhG-Genic Enhancers, Enh-Enhancers, ZNF/Rpts-ZNF genes and repeats, Het-Heterochromatin). (B) Same as A, but only considering Hi-C loci making contact with promoters of genes that are differentially expressed (DE).
(PDF)

S20 Fig Reciprocal enrichments of differential expression and differential contact. (A) Enrichment of inter-species differentially contacting (DC) loci in genes with corresponding differences in expression (DE) between the species. The proportion of DE genes that are significantly DC (y-axis) is shown across a range of DE FDRs (x-axis). Colors indicate different DC FDR thresholds, and dashed lines indicate the proportion of DC loci expected by chance alone. (B) P values of Chi-squared tests of the null that there is no difference in proportion of DC loci among DE genes (y-axis), shown for a range of DE FDRs (x-axis). In both panels, the DE genes overlapping Hi-C loci were chosen to have the minimum FDR supporting inter-species difference in expression. (C) Similar to Fig 6A, but using a weighted p-value combination technique [76] to integrate DC FDR across regions, instead of using the minimum FDR DC region. Once again, we observe enrichment of inter-species differentially expressed (DE) genes with corresponding differences in Hi-C contact frequencies (DC) between the species. The proportion of DC genes that are significantly DE (y-axis) is shown across a range of DC FDRs (x-axis). Colors indicate different DE FDR thresholds, and dashed lines indicate the proportion of DE genes expected by chance alone. (D) P values of Chi-squared tests of the null that there is no difference in proportion of DE genes among DC genes (y-axis), shown for a range of DC FDRs (x-axis).
(PDF)

S1 Table HOMER-called contacts, H21792. (TXT)

S2 Table HOMER-called contacts, H28126. (TXT)

S3 Table HOMER-called contacts, H28815. (TXT)

S4 Table HOMER-called contacts, H28834. (TXT)

S5 Table HOMER-called contacts, C3649. (TXT)

S6 Table HOMER-called contacts, C40300. (TXT)

S7 Table HOMER-called contacts, C3624. (TXT)

S8 Table HOMER-called contacts, C3651. (TXT)

S9 Table Orthology calling statistics. (XLSX)

S10 Table Differentially contacting (DC) regions. (TXT)

S11 Table Human consensus Arrowhead-inferred TADs. (TXT)

S12 Table Chimpanzee consensus Arrowhead-inferred TADs. (TXT)

S13 Table Human-Chimpanzee orthologous TAD coordinates. (TXT)

S14 Table ChromHMM genic enhancer annotation enrichments. (XLSX)

S15 Table ENCODE chromatin mark sources. (XLSX)

S16 Table Sample metadata. (XLSX)

S17 Table 10kb Arrowhead TAD inferences, H21792. (TXT)

S18 Table 10kb Arrowhead TAD inferences, H28126. (TXT)

S19 Table 10kb Arrowhead TAD inferences, H28815. (TXT)

S20 Table 10kb Arrowhead TAD inferences, H28834. (TXT)

S21 Table 10kb Arrowhead TAD inferences, C3649. (TXT)

S22 Table 10kb Arrowhead TAD inferences, C40300. (TXT)

S23 Table 10kb Arrowhead TAD inferences, C3624. (TXT)

S24 Table 10kb Arrowhead TAD inferences, C3651. (TXT)
