# Supplementary figures and images for: Reorganization of 3D genome structure may contribute to gene regulatory evolution in primates
Source: PLoS Genet. 2019 Jul 19;15(7):e1008278. doi: 10.1371/journal.pgen.1008278 (PMC6668850; doi:10.1371/journal.pgen.1008278)

**A**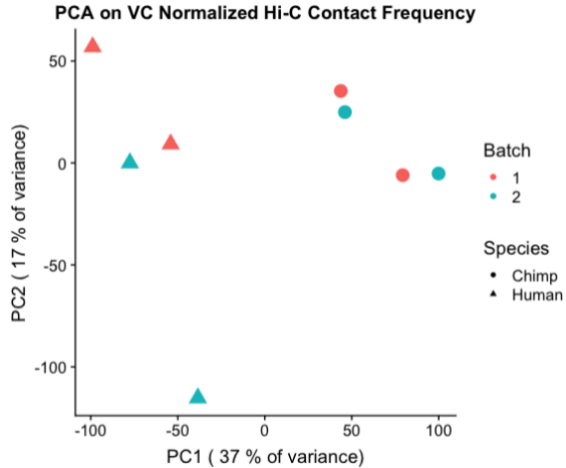**B**

Pairwise Pearson Correlation Clustering @ 10kb

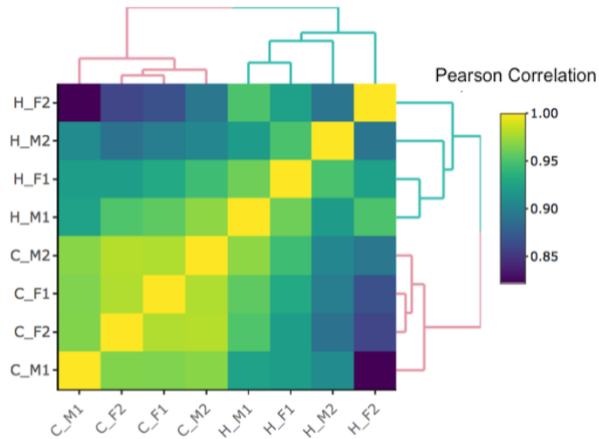

Supplement: S1 Fig — (A) Principal components analysis (PCA) of Juicer vanilla coverage (VC)-normalized interaction frequencies for the union of all contacts in humans (triangles) and chimpanzees (circles). PC1 is highly correlated with species (r = 0.89; P = 0.0004). (B) Unsupervised hierarchical clustering of the pairwise correlations (Pearson’s r2) of Juicer VC-normalized interaction frequencies at 10 kb resolution. The first letter in the labels demarcates the species (H for human and C for chimpanzee), and the following symbols indicate sex (male, M or female, F) and batch (1 or 2). (PDF) [file pgen.1008278.s001.pdf]

**A**

### Contact Frequency Differences

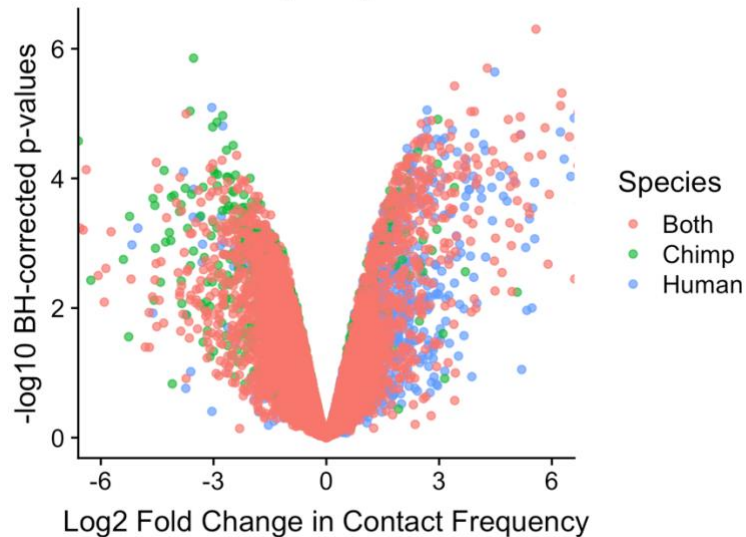**B**

### Volcano Plots by Chromosome, VC Normalization

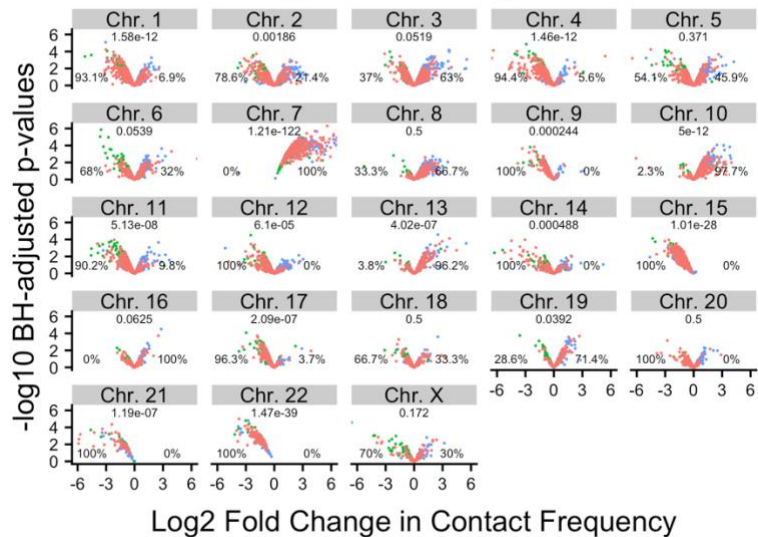

Supplement: S2 Fig — (A) Volcano plot of log2 fold change in contact frequency between humans and chimpanzees (x-axis) against Benjamini-Hochberg FDR (y-axis), after filtering non-orthologous regions. Data are colored by the species in which the contact was originally identified as significant. (B) Per-chromosome volcano plot using the same legend as in A. P-values provided for a binomial test of the null that inter-species differences in contact frequencies are evenly distributed. The percentage of contacts with significant higher frequency in each species is noted. Of note is that many of the same chromosomal asymmetries in contact strength observed here are in the same chromosomes as those observed in the HOMER-normalized data (Fig 2). (PDF) [file pgen.1008278.s002.pdf]

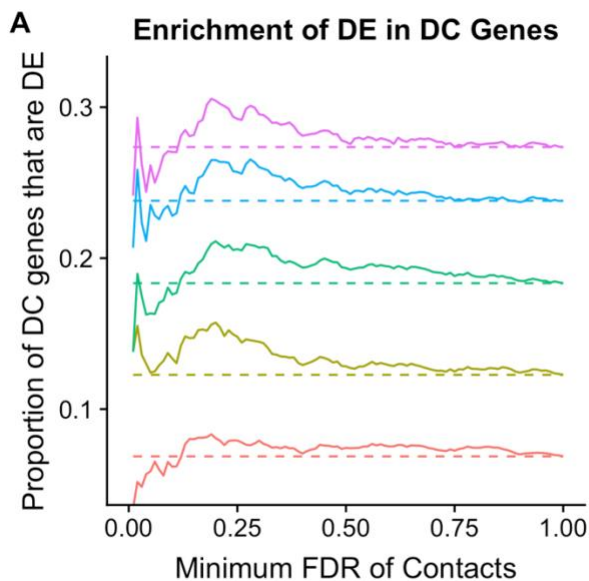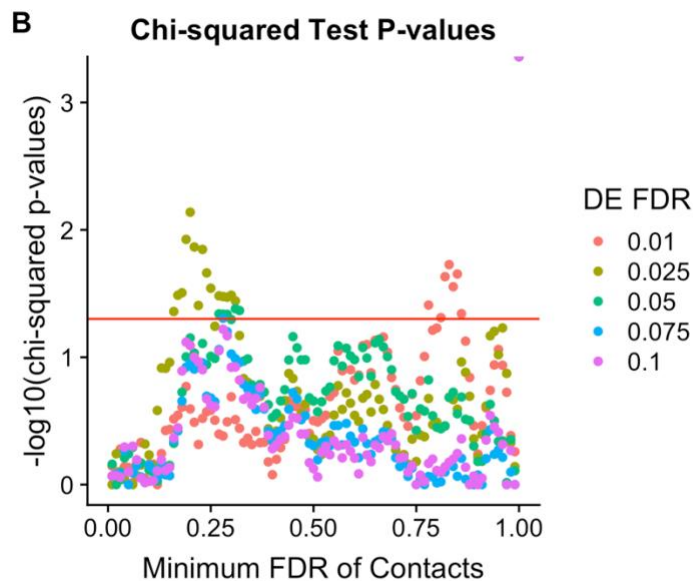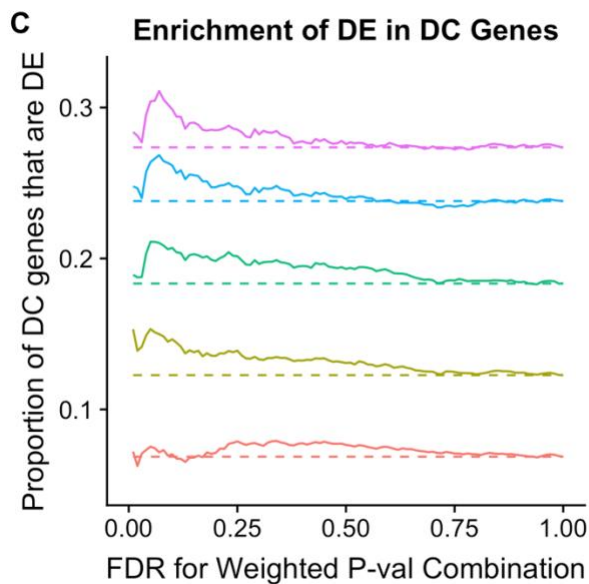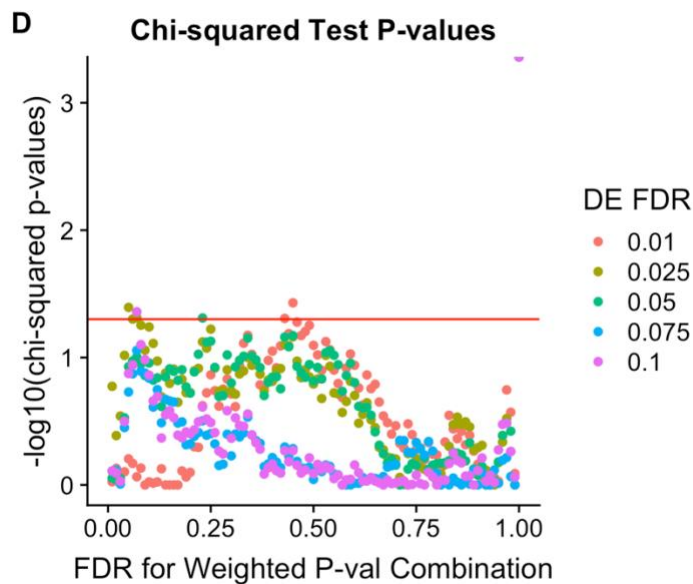

Supplement: S3 Fig — (A) Enrichment of inter-species differentially expressed (DE) genes with corresponding differences in Hi-C contact frequencies (DC) between the species. The proportion of DC genes that are significantly DE (y-axis) is shown across a range of DC FDRs (x-axis). Colors indicate different DE FDR thresholds, and dashed lines indicate the proportion of DE genes expected by chance alone. (B) P values of Chi-squared tests of the null that there is no difference in proportion of DE genes among DC genes (y-axis), shown for a range of DC FDRs (x-axis). In both panels, DC regions were chosen to have the minimum FDR supporting inter-species difference in contact frequency. (C) Same as A, but this time, a weighted p-value combination technique [76] was used to integrate each Hi-C bin’s DC FDR across all of its contacts. (D) Same as B, but for the weighted p-value combination instead of the minimum FDR contact. (PDF) [file pgen.1008278.s003.pdf]

**A**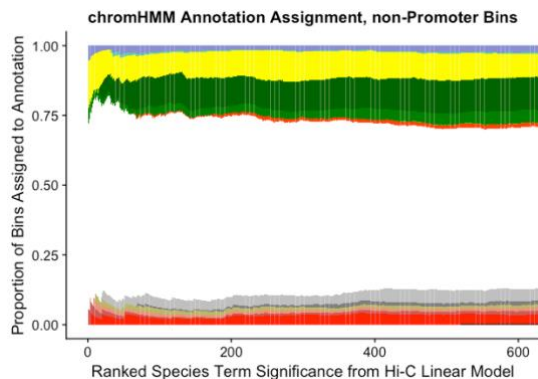**B**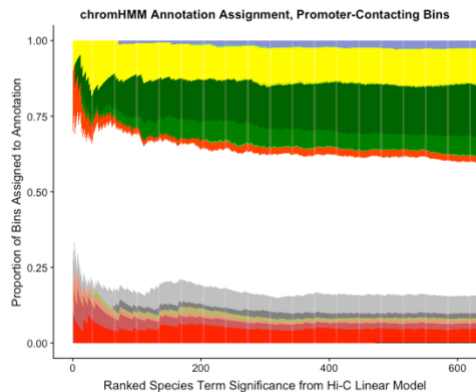**C**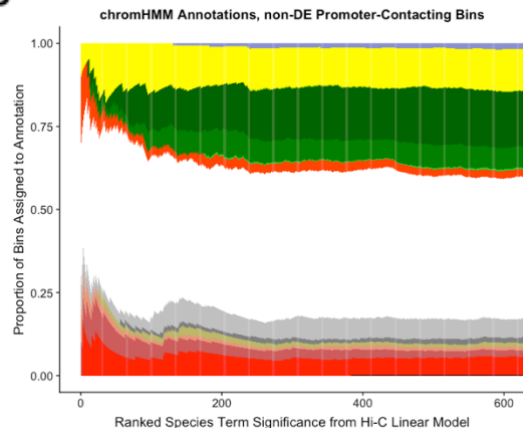**D**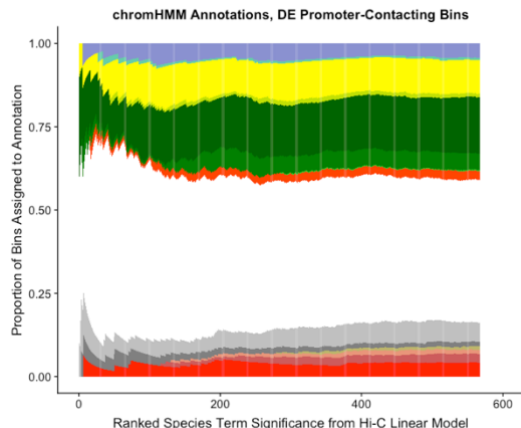

### Annotations

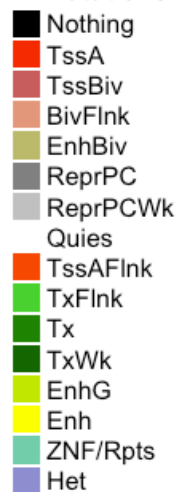

Supplement: S4 Fig — (A) Hi-C loci that do not make contact with promoters are ranked in order of decreasing DC FDR (x-axis). The y-axis shows cumulative proportion of chromHMM annotation assignments for all Hi-C loci at the given FDR or lower. (TssA-Active TSS, TSSBiv-Bivalent/Poised TSS, BivFlnk-Flanking Bivalent TSS/Enh, EnhBiv-Bivalent Enhancer, ReprPC-Repressed PolyComb, ReprPCWk-Weak Repressed PolyComb, Quies-Quiescent/Low, TssAFlnk-Flanking Active TSS, TxFlnk-Transcription at gene 5’ and 3’, Tx-Strong transcription, TxWk-Weak transcription, EnhG-Genic Enhancers, Enh-Enhancers, ZNF/Rpts-ZNF genes and repeats, Het-Heterochromatin). (B) Same as A, but only considering Hi-C loci making contact with promoter bins. (C) Same as B, but only considering Hi-C loci making contact with promoters of genes that are not differentially expressed (DE). (D) Same as C, but only considering Hi-C loci making contact with promoters of genes that are differentially expressed (DE). (PDF) [file pgen.1008278.s004.pdf]

## Effect of Contact on Expression Divergence in DE genes

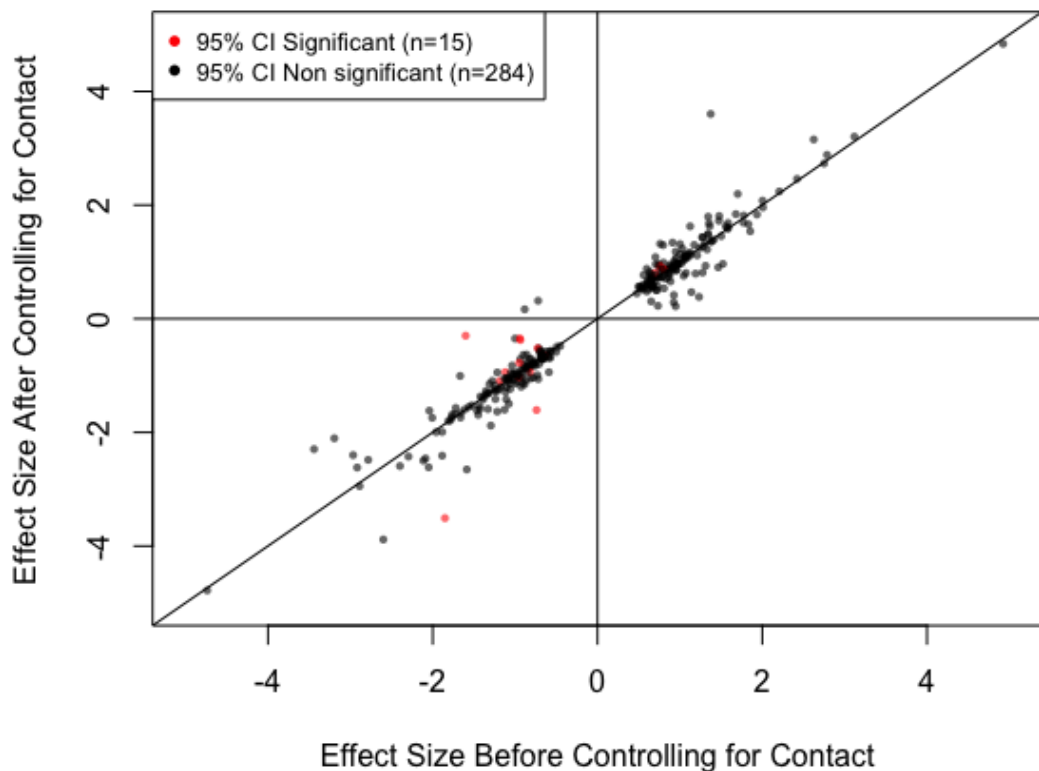

Supplement: S6 Fig — Plot of the species effect size in DE genes between models before (x-axis) and after (y-axis) conditioning on contact frequency. The Monte Carlo test of significance was used to construct the 95% confidence interval and evaluate the significance of the indirect effect (species’ effect on expression mediated through contact). Amongst DE genes, 5% (15/299) of genes showed a statistically significant effect of Hi-C contacts on expression levels (i.e. their 95% confidence interval does not include zero). (PDF) [file pgen.1008278.s006.pdf]

**A**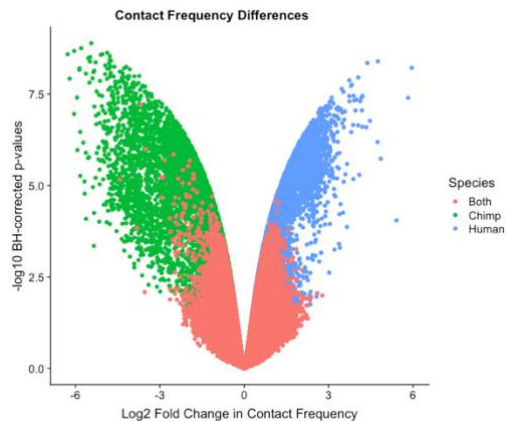**C**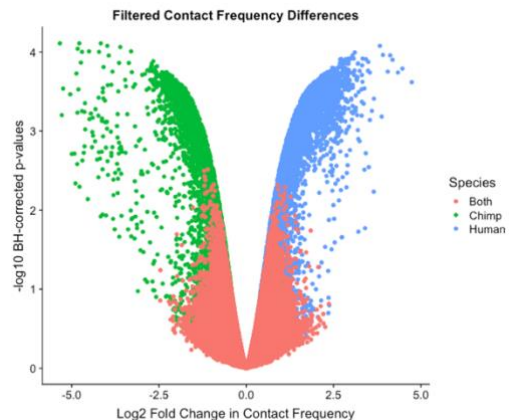**B**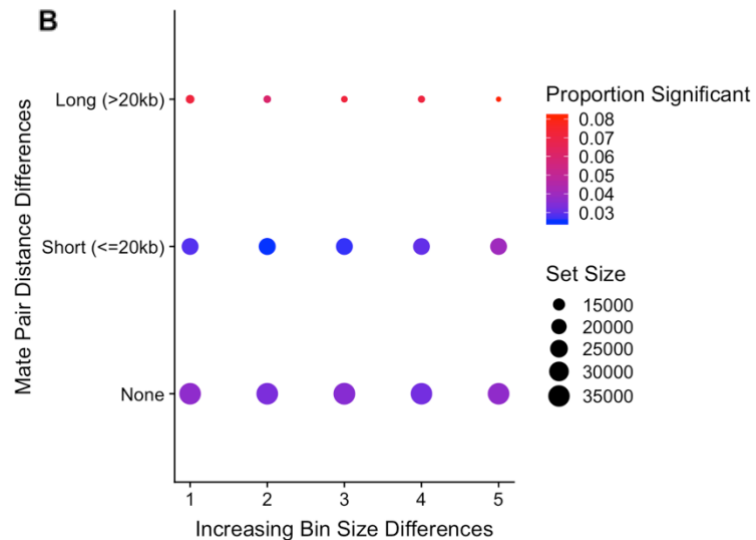

Supplement: S9 Fig — (A) Volcano plot of log2 fold change in contact frequency between humans and chimpanzees (x-axis) against Benjamini-Hochberg FDR (y-axis). This plot shows data only filtered for independent discovery in at least 4 individuals. Data are colored by the species in which the contact was originally identified as significant. (B) Scatter plot of sets of Hi-C contacts, with proportion of contacts significant in our linear modeling of interaction frequency shown based on color. Contacts are binned by mate-pair distance differences (y-axis) and bin size differences (x-axis). Circle size is proportional to the size of the set of Hi-C contacts falling into each criteria. Red indicates that the data were filtered out after this step, and blue/purple indicates that the data were retained for further analysis. (C) Volcano plot as in A, but after removing contacts with large mate-pair distance differences across the species. (PDF) [file pgen.1008278.s009.pdf]

**A**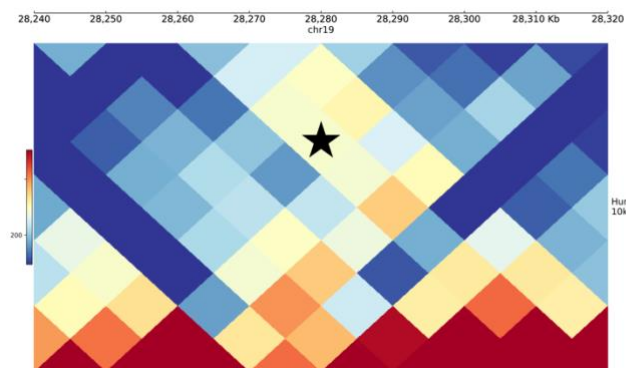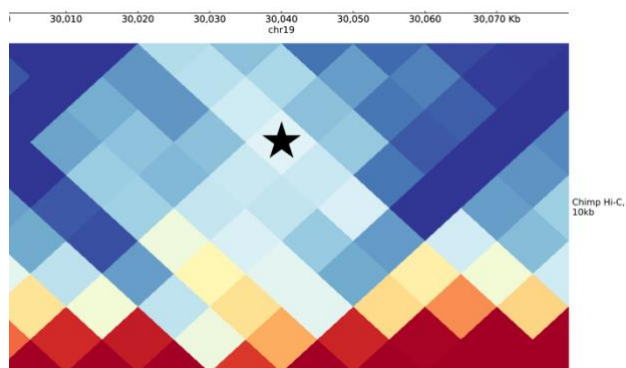**B**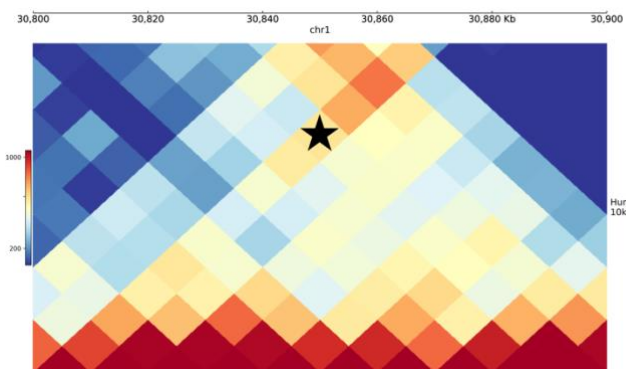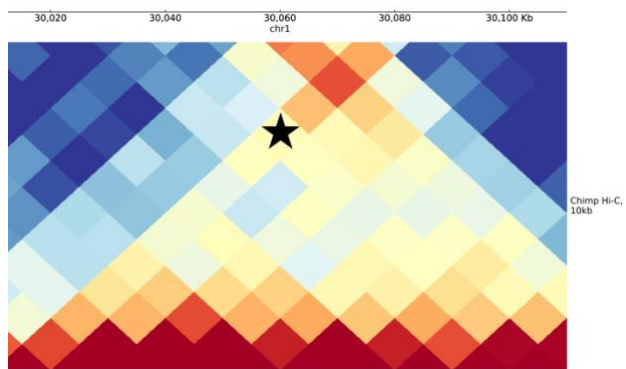**C**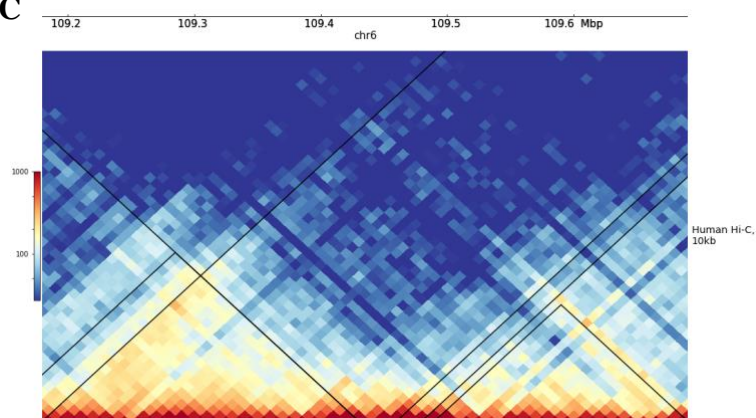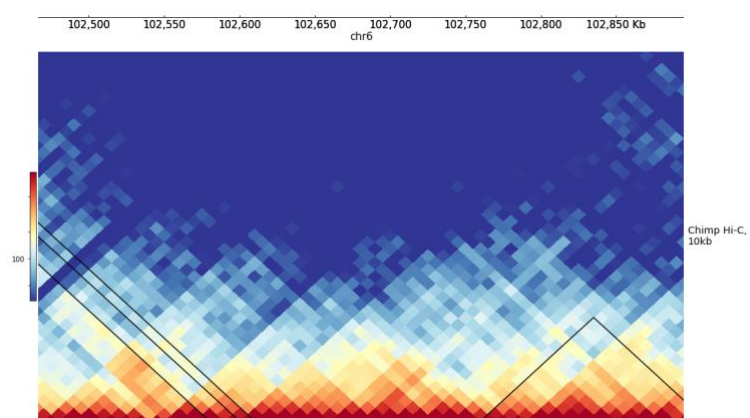**D**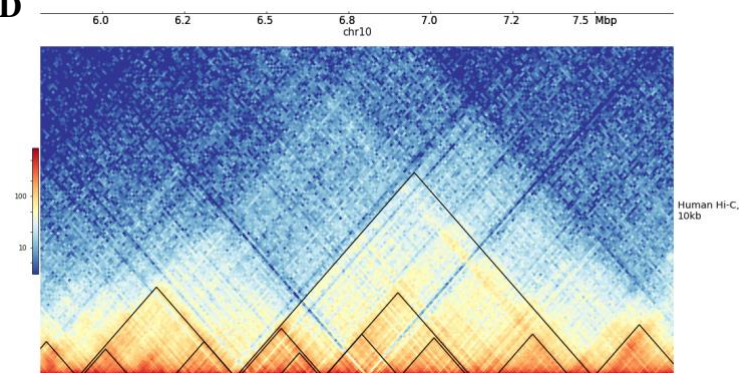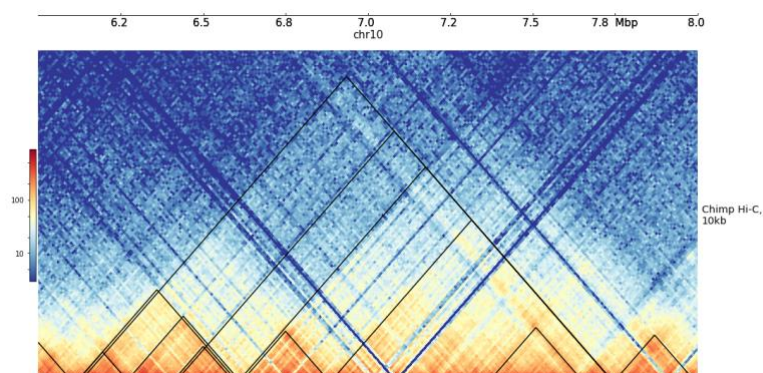

**E**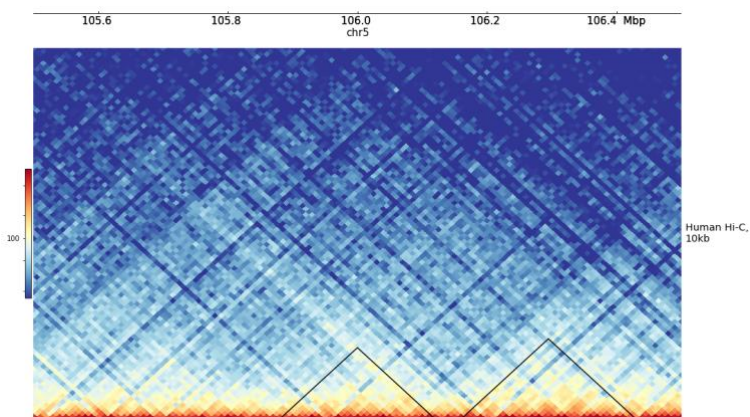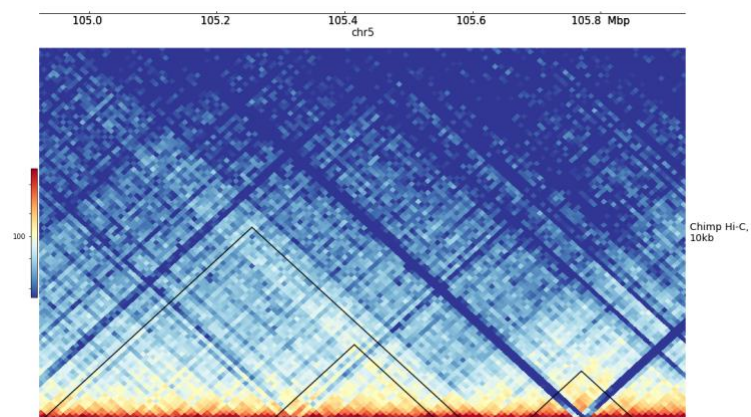**F**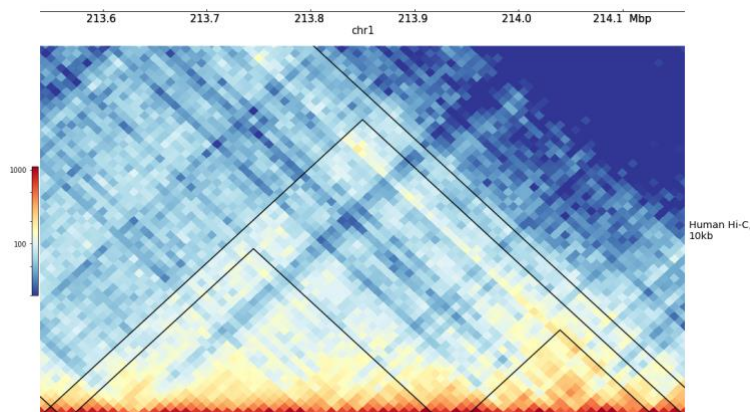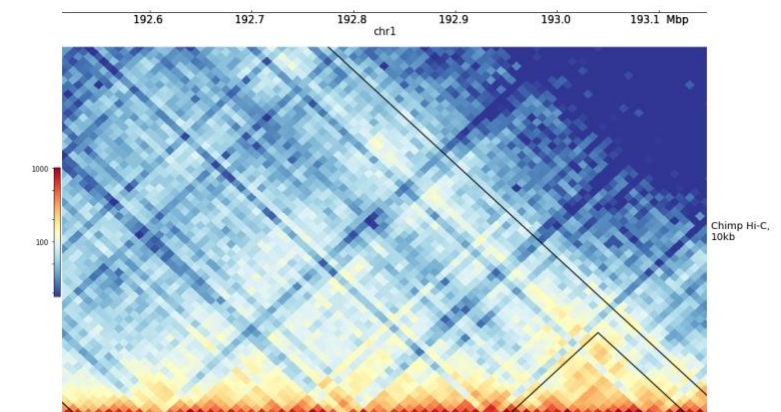**G**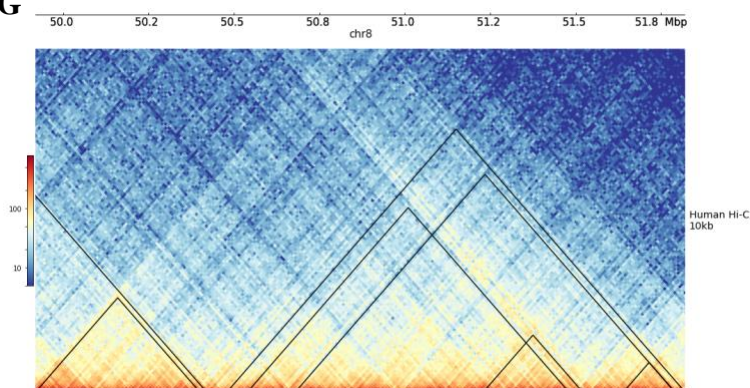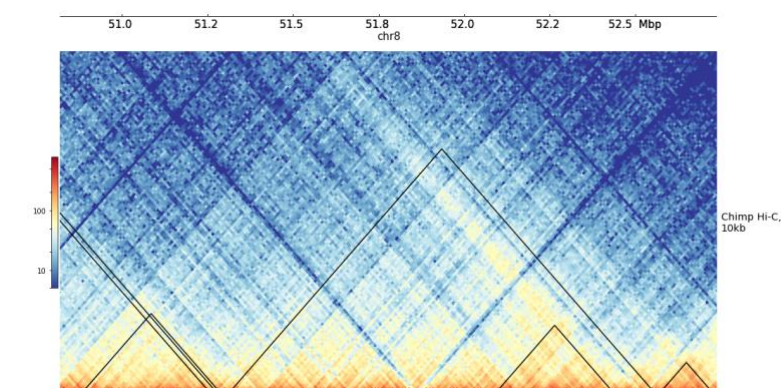**H**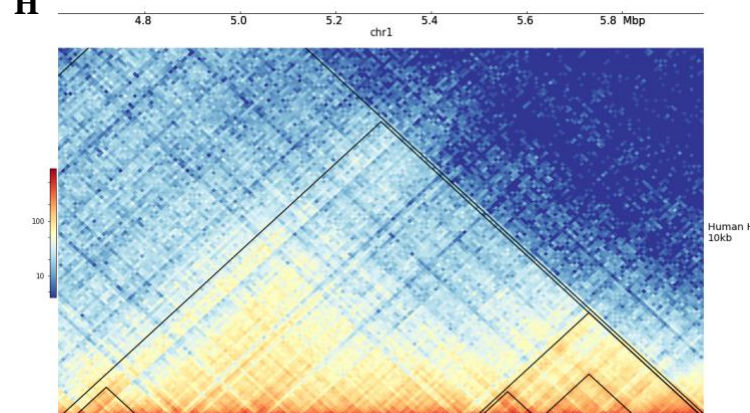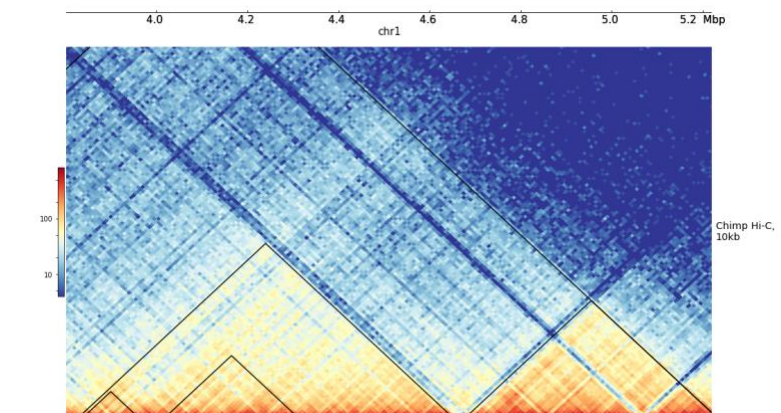

Supplement: S10 Fig — (A) PyGenomeTracks plots [84] of a chromosome 19 interaction between bins 80 kb away for human (left panel) and chimpanzee (right panel). The bin pair tested is indicated by a black star, and was found to be DC between species. (B) Same as A, but for a conserved (non-DC) interaction on chromosome 1 separated by 100kb. (C-H) Examples of contact maps (created with PyGenomeTracks [84]) and Arrowhead-inferred TAD structures (black lines) in humans (left) and chimpanzees (right), across a number of different chromosomes. In most examples, inference based on the algorithm indicates shared and species-specific domains, yet these are difficult to ascertain based on visual inspection, as discussed. (PDF) [file pgen.1008278.s010.pdf]

**A**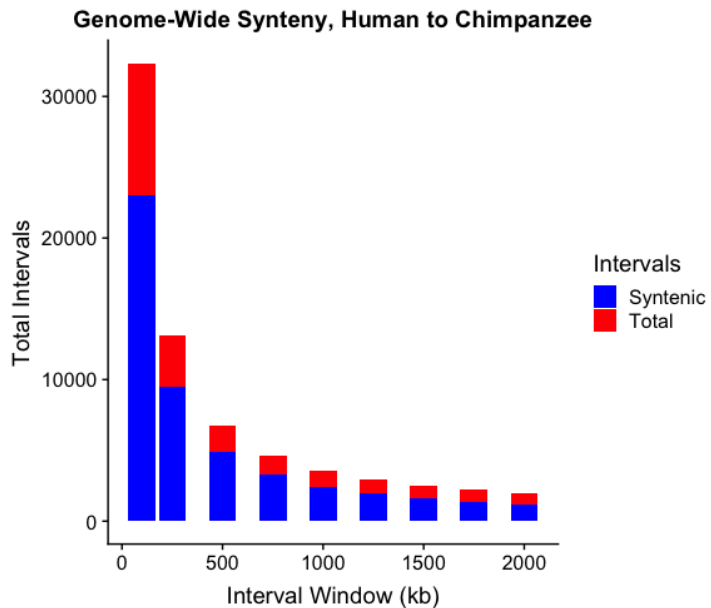**B**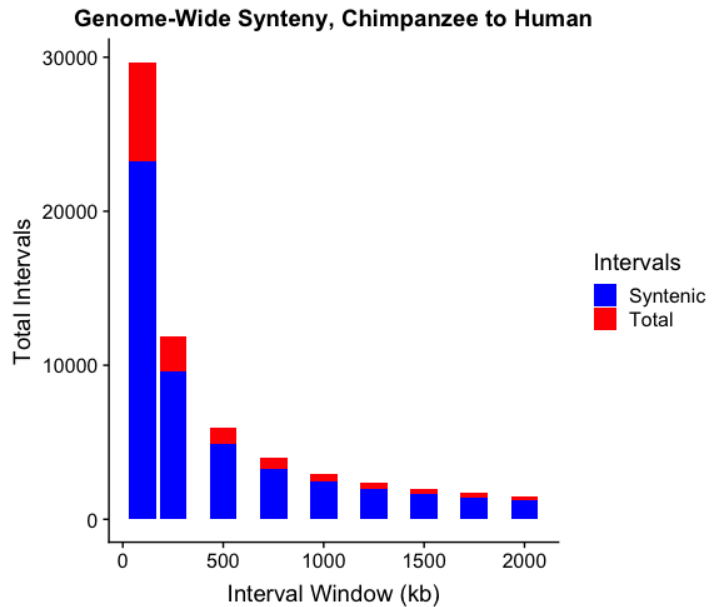

Supplement: S11 Fig — (A) Across different window sizes (x-axis) for a genome-wide tiling of hg38, we plotted the number of total and syntenic linear intervals (y-axis), identified using the reciprocal best hits liftOver method [104, 105] we employed throughout the paper. (B) Same as A, but for a genome-wide tiling of panTro5. (PDF) [file pgen.1008278.s011.pdf]

**A**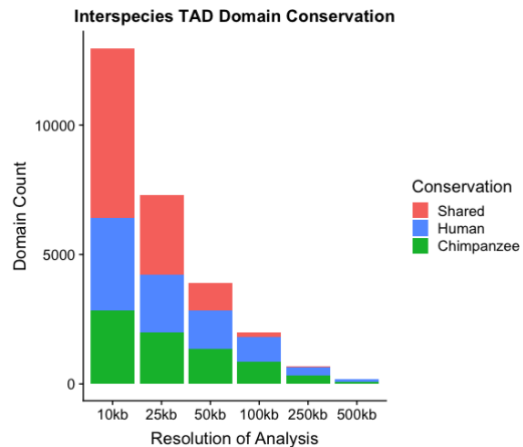**B**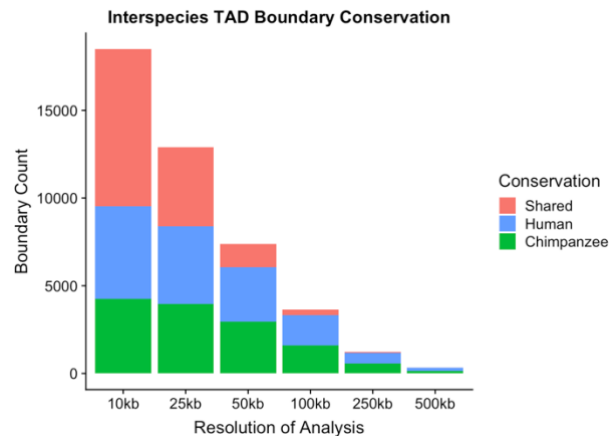**C**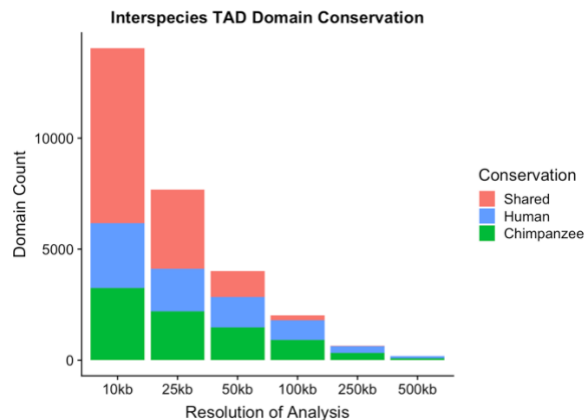**D**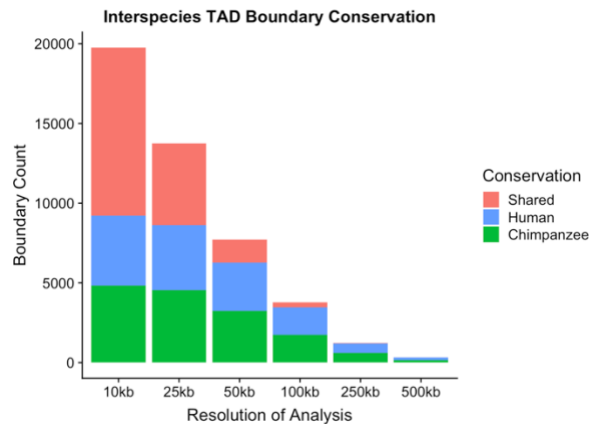

Supplement: S13 Fig — (A) Across different resolutions (x-axis), we plotted the number of shared and species-specific domains (y-axis) identified with Arrowhead [62] using the consensus map from each species. To call a domain as conserved here, we required that the Euclidean distance between the domain across species be less than the minimum of 50kb or 50% the length of the TAD, based on the conservation calling method employed by Rao et al [45]. Results are highly similar to those seen in Fig 4A. (B) Same as A, but for TAD boundaries instead of the domains themselves. Boundaries were defined as 15 kb flanking regions at the edges of inferred Arrowhead domains. In this case, conservation was called if there was any base pair overlap between boundaries. Unlike in Fig 4B, boundaries were merged before calling conservation, in order to find unique boundary elements. This difference in analysis paradigms could have important consequences with a nested TAD caller such as Arrowhead [62], but results are highly similar to those seen in Fig 4B. (C) Same as A, but this time, performed on “high-density consensus” Hi-C maps that have been mapped to the hg38 and panTro6 genomes (rather than panTro5). Results are highly similar despite the improvement in genome quality build. (D) Same as B, but this time, on the hg38 and panTro6 genome assemblies. (PDF) [file pgen.1008278.s013.pdf]

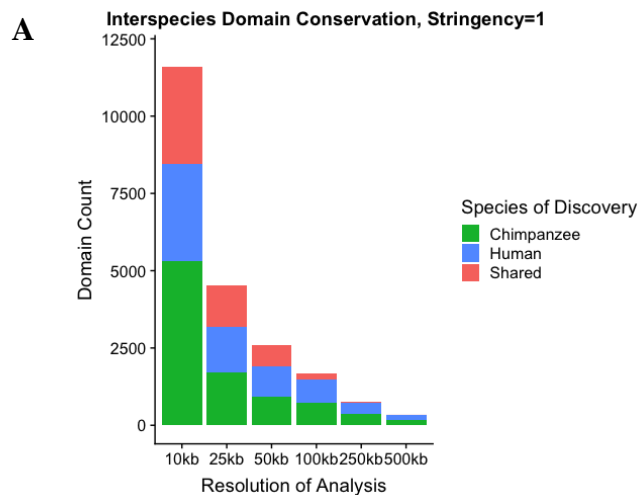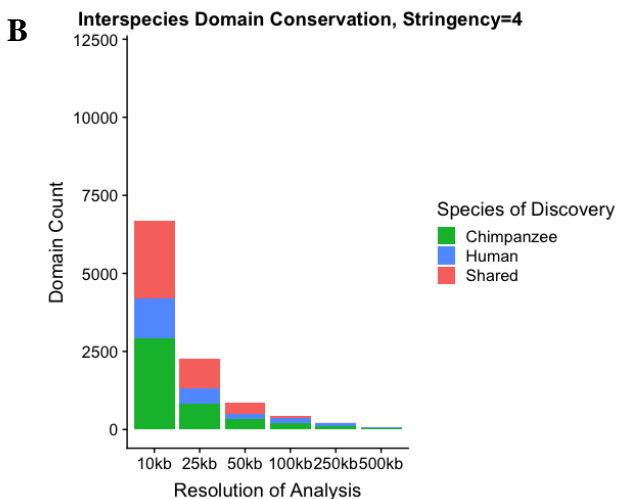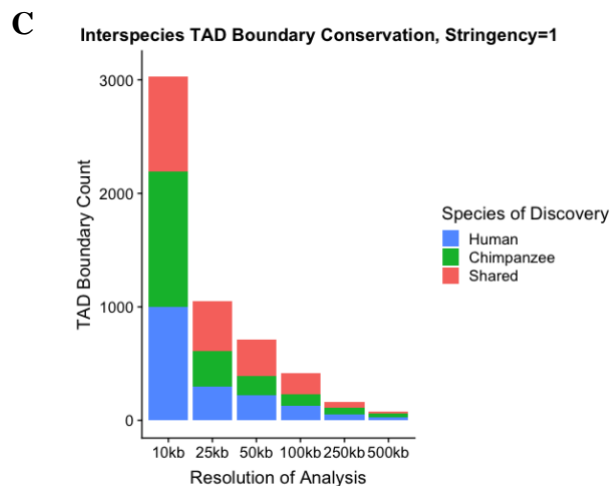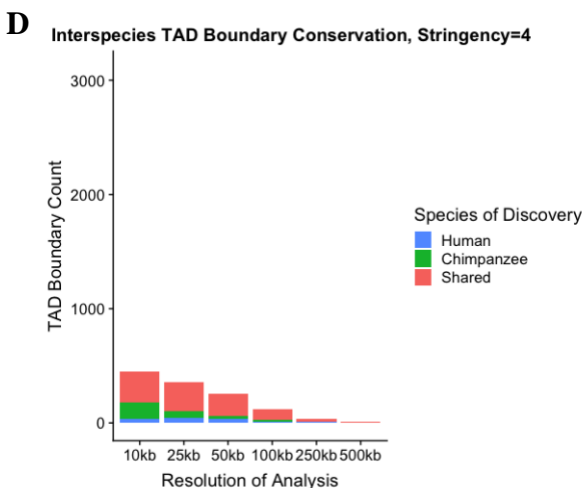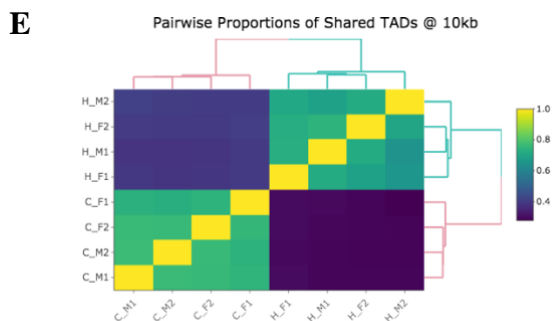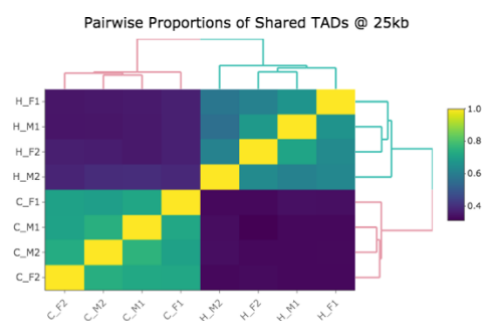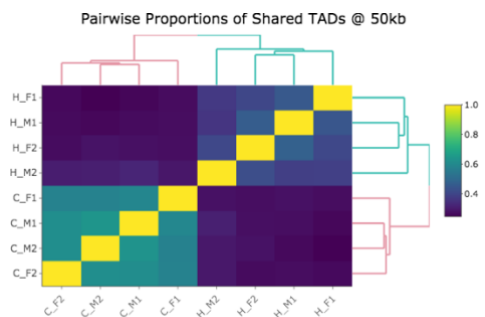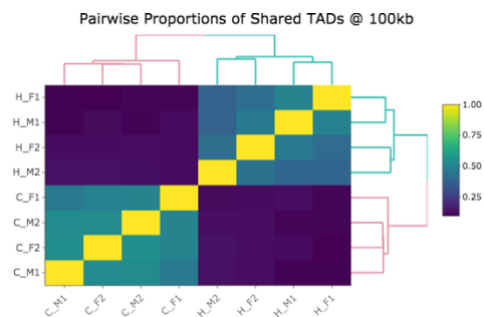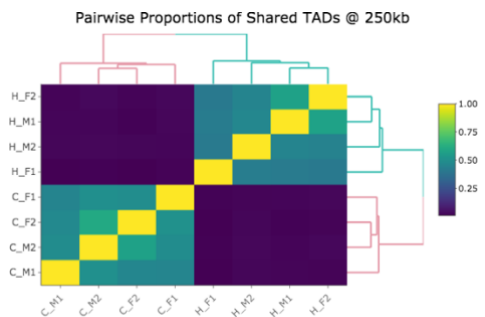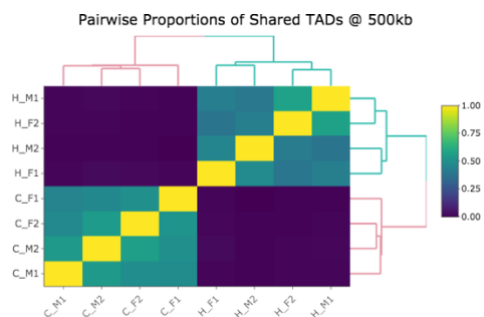

**F**

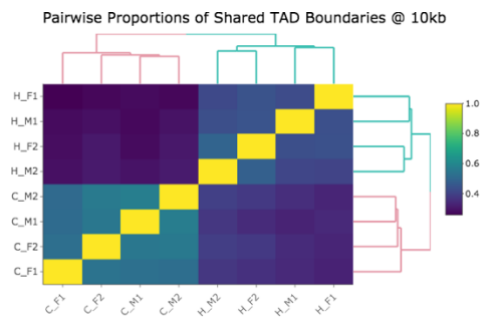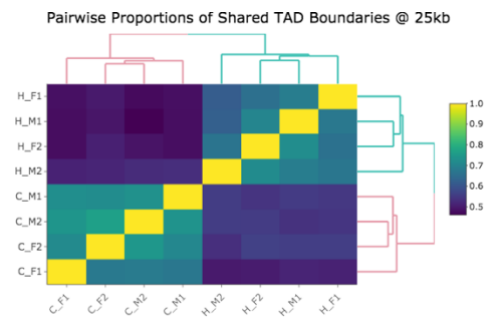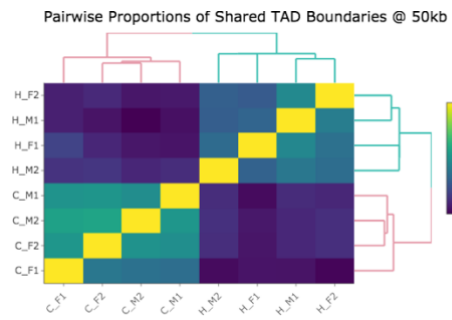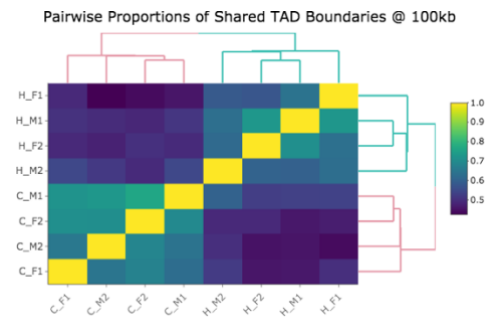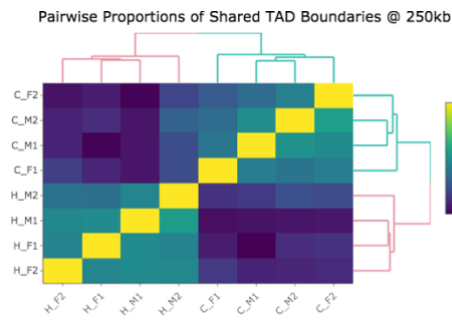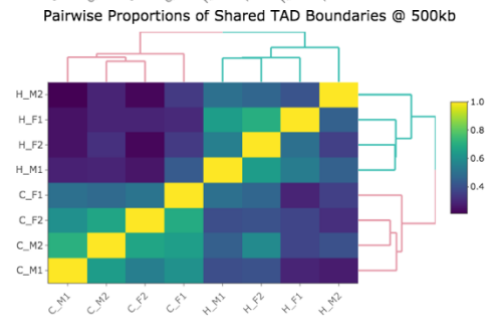

Supplement: S14 Fig — (A) Across different resolutions (x-axis), we plotted the number of shared and species-specific domains (y-axis) identified with TopDom [68] on HOMER-normalized Hi-C maps from each individual. We called domain conservation here based on the method of Rao et al. [45] (highly similar results were observed with our 90% reciprocal overlap method, described in the text and available in the github repository associated with the paper). Domain count values represent the average interspecies sharing across all individuals, with no filtering for domain robustness (that is, assessing all domains discovered and orthologously mappable). Under this analysis paradigm we observe relatively low sharing across species (maximum of 30% at 25 kb). (B) Same as A, but this time, only considering TADs that were found across all 4 individuals within either one of the species (fixed TADs). Restricting to this subset increases the maximum percentage of conservation to 42% at 25 kb resolution, although the set of TADs being examined is much smaller. (C) Same as A, but for TopDom [68] boundary inferences instead of domains. We considered boundaries shared between individuals if they had any overlap. (D) Same as B, but for boundaries instead of domains (i.e. considering only boundaries fixed within species). Here, the highest estimate of conservation we obtain is 76% of boundaries conserved across species at 50 kb resolution. (E) Unsupervised hierarchical clustering of the pairwise proportions of shared TADs between all individuals in our study at a variety of resolutions, using the Rao et al. [45] methodology for calling conservation. The first letter in the labels demarcates the species (H for human and C for chimpanzee), and the following symbols indicate sex (male, M or female, F) and batch (1 or 2). Heatmaps are not necessarily symmetric because different numbers of TADs were discovered in different individuals; rows represent an individual’s shared proportion of TADs (individual total) wi [file pgen.1008278.s014.pdf]

## Correlation b/t RPKM Expression and Hi-C Contact Frequency

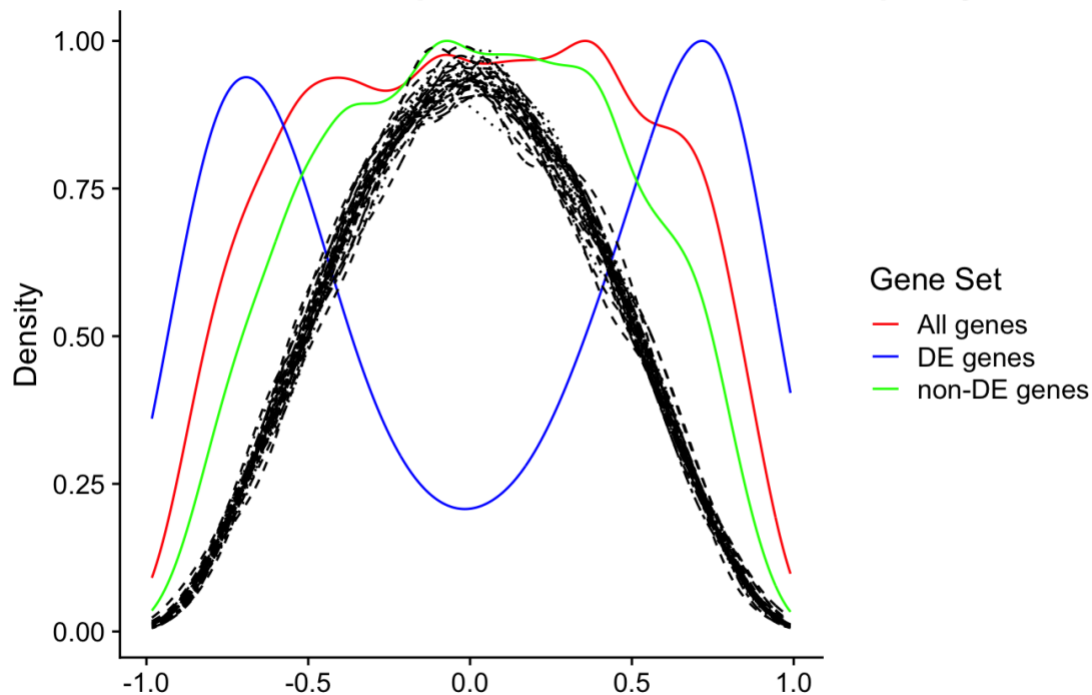

Supplement: S15 Fig — Density of Pearson correlations between RPKM expression values and log2 HOMER-normalized contact frequencies across all 8 individuals. Solid lines indicate different sets of the observed data and dotted lines represent 10 permutations of the data. The Hi-C contact frequency chosen is that with the minimum FDR from linear modeling of contact frequency on species (see main text). The strong bimodal distribution of correlations between expression and contact suggests many instances where a contact difference between the species can lead to an increase (enhancer) or decrease (suppressor) of expression in the species where the contact is stronger. (PDF) [file pgen.1008278.s015.pdf]

## Effect of Contact on Expression Divergence in DE genes

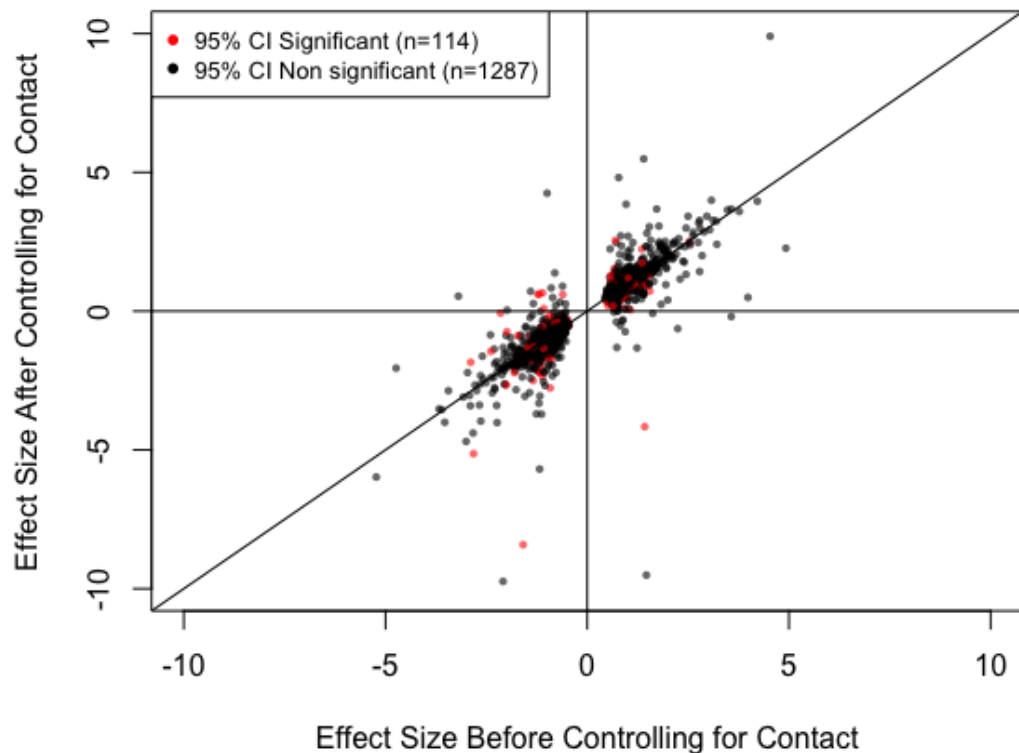

Supplement: S16 Fig — Plot of the species effect size in DE genes between models before (x-axis) and after (y-axis) conditioning on contact frequency. The Monte Carlo test of significance was used to construct the 95% confidence interval and evaluate the significance of the indirect effect (species’ effect on expression mediated through contact). Amongst DE genes, 8% (116/1401) of genes showed a statistically significant effect of Hi-C contacts on expression levels (i.e. their 95% confidence interval does not include zero). (PDF) [file pgen.1008278.s016.pdf]

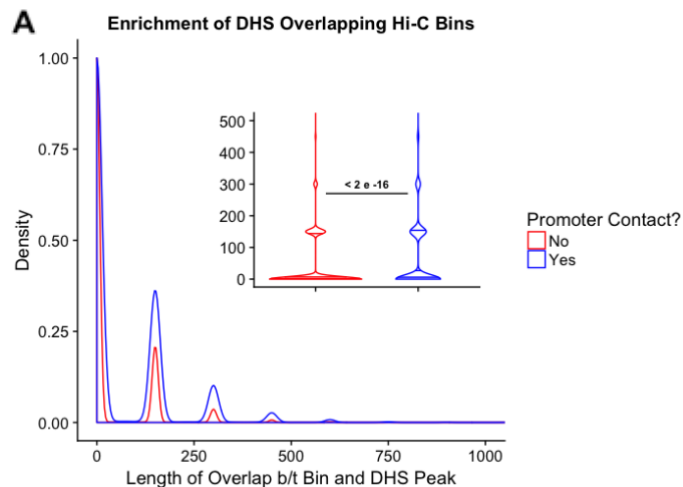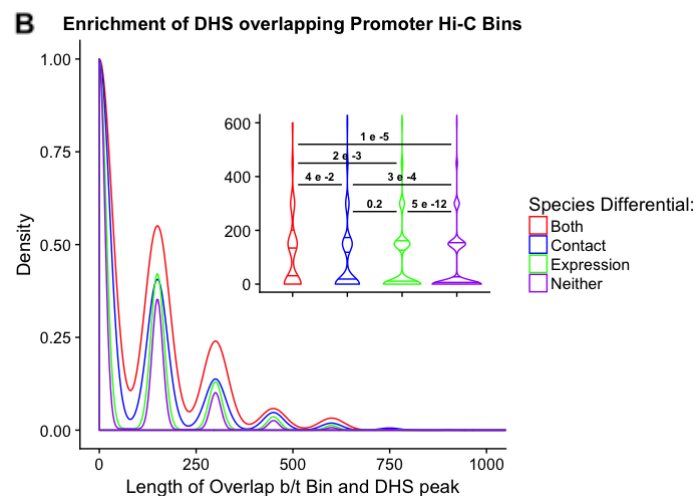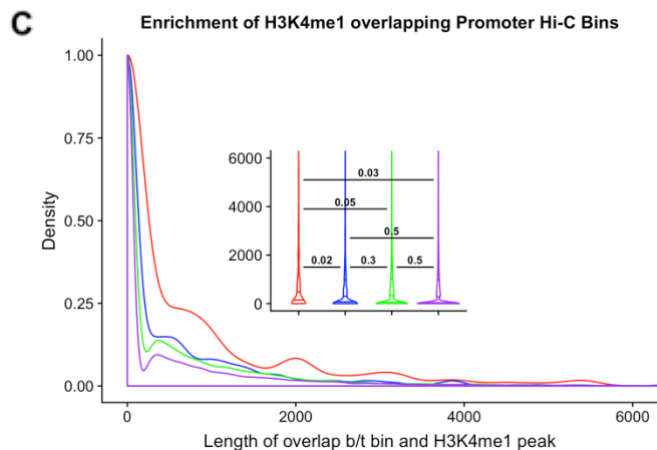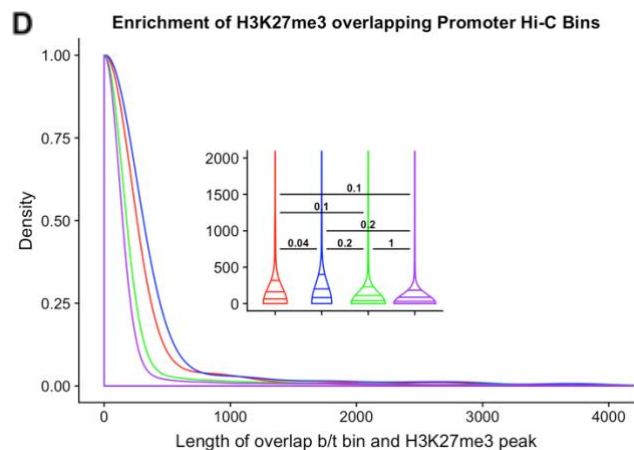

Supplement: S18 Fig — (A) Density distribution of the base pair overlap between DHS peaks downloaded from ENCODE and our Hi-C loci. Plot is split between Hi-C loci that contact a promoter and those that do not. Inlay is a violin plot of the same distributions, with lines and numbers indicating pairwise t-tests of the mean, and their corresponding significance levels. (B) Density plot similar to A, but only considering Hi-C loci involved in contact with a promoter, and separating contacts into 4 classes, indicated by color: those that show differential contact between species, those that show differential expression between species, those that show both, and those that show neither. We used pairwise t-tests to compare differences in the mean overlap among the four classes of Hi-C loci. (C) Same as in B, but for the active histone mark H3K4me1. (D) Same as in B and C, but for the repressive histone mark H3K27me3. (PDF) [file pgen.1008278.s018.pdf]

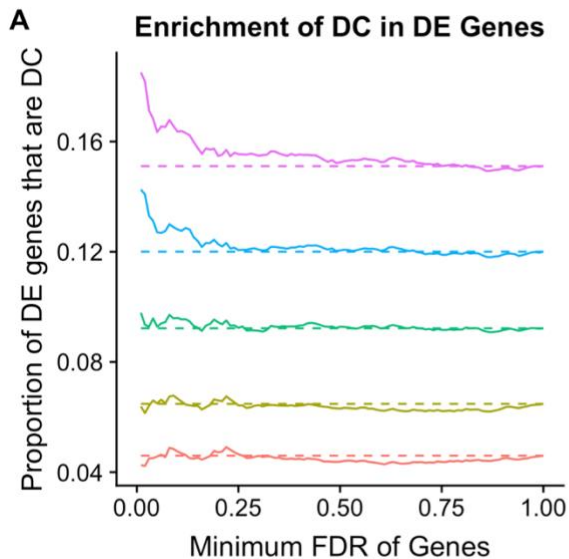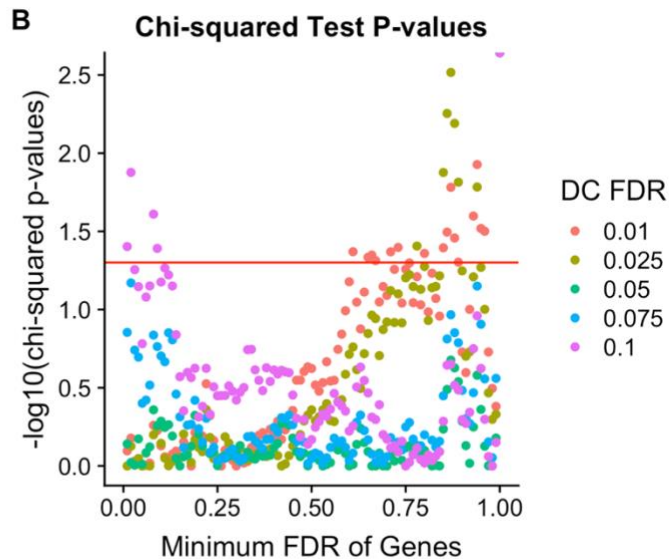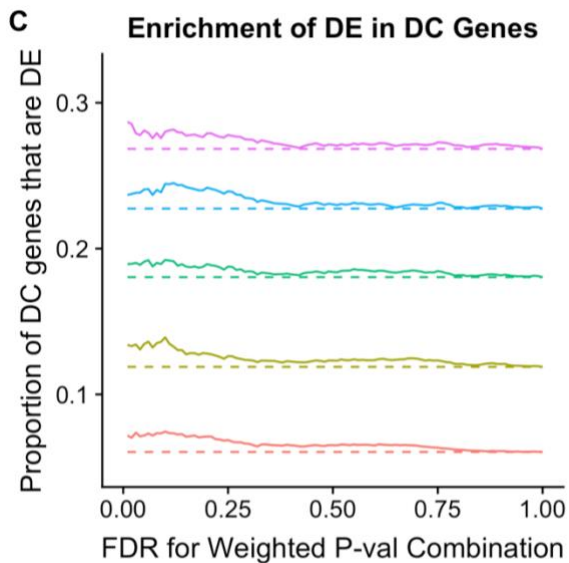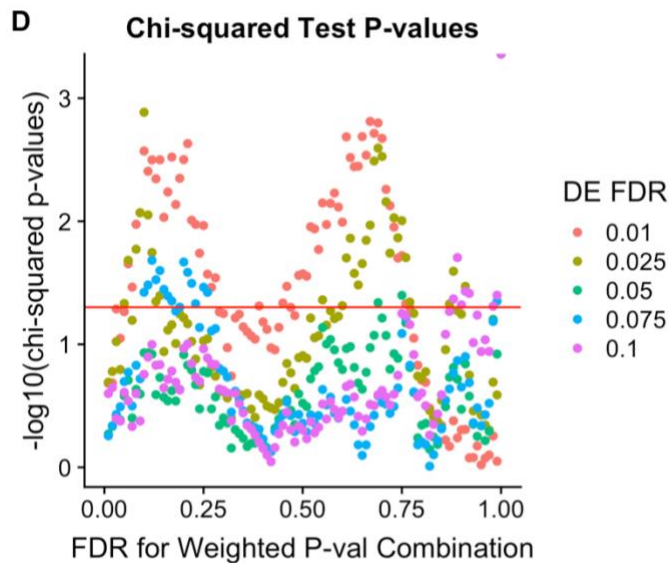

Supplement: S20 Fig — (A) Enrichment of inter-species differentially contacting (DC) loci in genes with corresponding differences in expression (DE) between the species. The proportion of DE genes that are significantly DC (y-axis) is shown across a range of DE FDRs (x-axis). Colors indicate different DC FDR thresholds, and dashed lines indicate the proportion of DC loci expected by chance alone. (B) P values of Chi-squared tests of the null that there is no difference in proportion of DC loci among DE genes (y-axis), shown for a range of DE FDRs (x-axis). In both panels, the DE genes overlapping Hi-C loci were chosen to have the minimum FDR supporting inter-species difference in expression. (C) Similar to Fig 6A, but using a weighted p-value combination technique [76] to integrate DC FDR across regions, instead of using the minimum FDR DC region. Once again, we observe enrichment of inter-species differentially expressed (DE) genes with corresponding differences in Hi-C contact frequencies (DC) between the species. The proportion of DC genes that are significantly DE (y-axis) is shown across a range of DC FDRs (x-axis). Colors indicate different DE FDR thresholds, and dashed lines indicate the proportion of DE genes expected by chance alone. (D) P values of Chi-squared tests of the null that there is no difference in proportion of DE genes among DC genes (y-axis), shown for a range of DC FDRs (x-axis). (PDF) [file pgen.1008278.s020.pdf]
