# Supplementary material for: Reorganization of 3D genome structure may contribute to gene regulatory evolution in primates
Source: PLoS Genet. 2019 Jul 19;15(7):e1008278. doi: 10.1371/journal.pgen.1008278 (PMC6668850; doi:10.1371/journal.pgen.1008278)
Supplement: S5 Fig — (A) Density plot of the base pair overlap between different classes of Hi-C contact loci and H3K27ac. Histone mark data were obtained from ENCODE in experiments carried out in human iPSCs. We grouped contacts into 4 classes, indicated by color: those that show differential contact between species, those that show differential expression between species, those that show both, and those that show neither. We used pairwise t-tests to compare differences in the mean overlap among the four classes of Hi-C loci. Unlike in the HOMER-normalized data, we do not observe statistically significant differences in overlaps with H3K27ac between different locus classes. This may reflect the previous observation that the hiccups algorithm for assigning statistical significance of loops in Hi-C data is much more conservative than HOMER’s significance calling method [86]. (B) Same as A, but performed on H3K4me3 data obtained from ENCODE, collected in hESCs. (C) Same as A and B, but performed on H3K4me1 data obtained from ENCODE, collected in human iPSCs. (D) Same as A, B, and C, but performed on H3K27me3 data obtained from ENCODE, collected in human iPSCs. (PDF) [file pgen.1008278.s005.pdf]

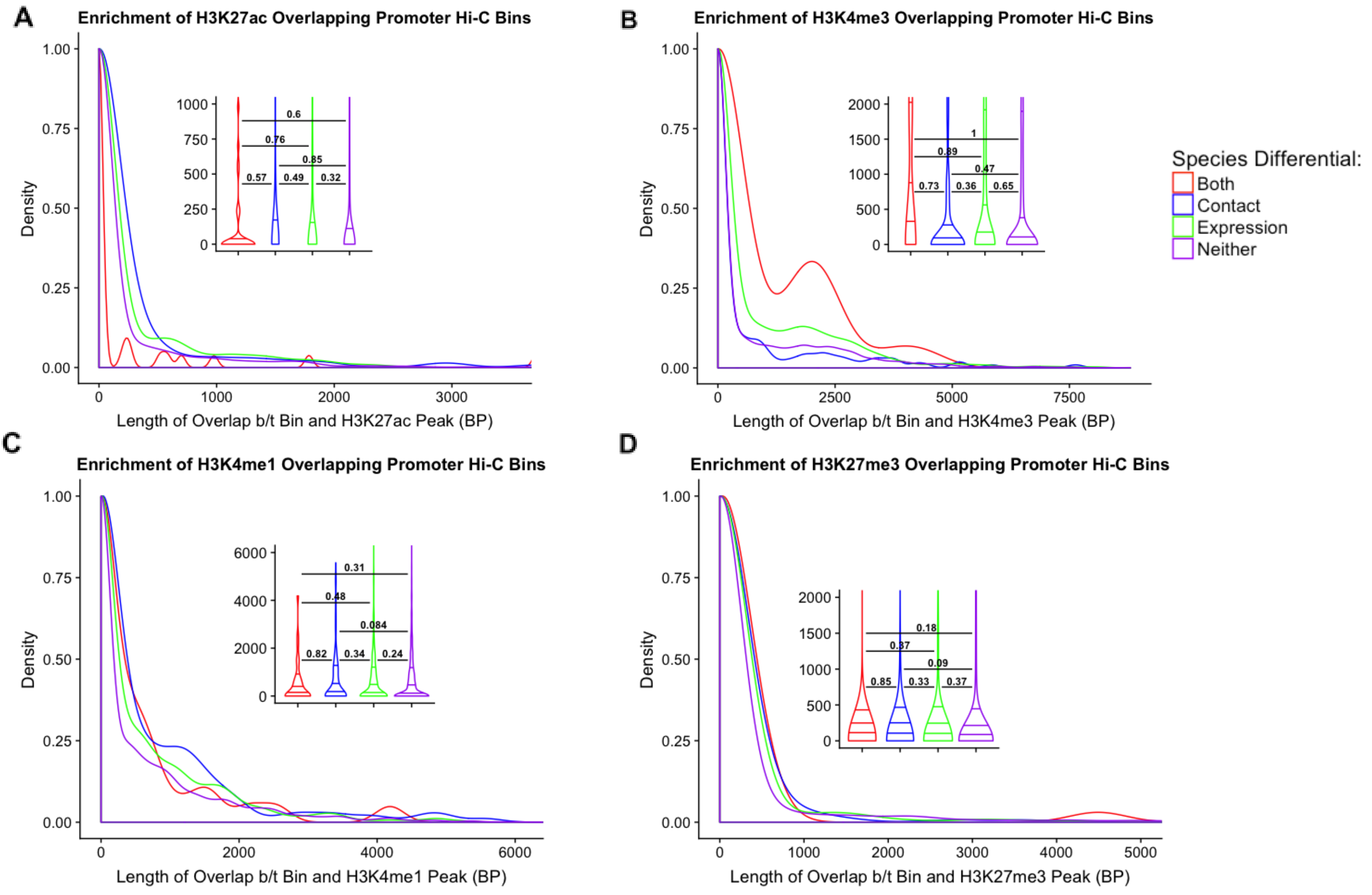
A
Enrichment of H3K27ac Overlapping Promoter Hi-C Bins
Density
Length of Overlap b/t Bin and H3K27ac Peak (BP)
0.6
0.76
0.85
0.57
0.49
0.32
B
Enrichment of H3K4me3 Overlapping Promoter Hi-C Bins
Density
Length of Overlap b/t Bin and H3K4me3 Peak (BP)
1
0.89
0.47
0.73
0.36
0.65
Species Differential:
Both
Contact
Expression
Neither
C
Enrichment of H3K4me1 Overlapping Promoter Hi-C Bins
Density
Length of Overlap b/t Bin and H3K4me1 Peak (BP)
0.31
0.48
0.084
0.82
0.34
0.24
D
Enrichment of H3K27me3 Overlapping Promoter Hi-C Bins
Density
Length of Overlap b/t Bin and H3K27me3 Peak (BP)
0.18
0.37
0.09
0.85
0.33
0.37
