# Supplementary material for: Reorganization of 3D genome structure may contribute to gene regulatory evolution in primates
Source: PLoS Genet. 2019 Jul 19;15(7):e1008278. doi: 10.1371/journal.pgen.1008278 (PMC6668850; doi:10.1371/journal.pgen.1008278)
Supplement: S7 Fig — (A) Boxplots of variance in contact frequency across all 8 individuals on the y-axis, binned by the number of individuals in which an interaction is independently called significant on the x-axis. (B) Same as A, but zoomed in on the y-axis to visualize finer-scale variation. (PDF) [file pgen.1008278.s007.pdf]

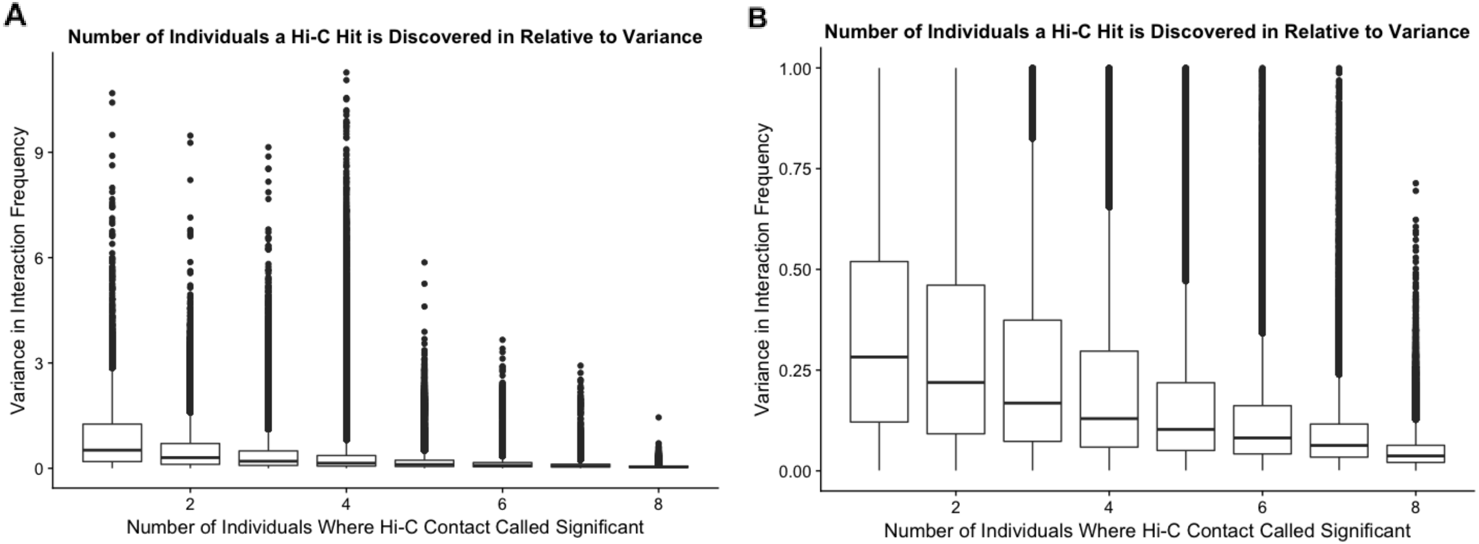
A
Number of Individuals a Hi-C Hit is Discovered in Relative to Variance
Variance in Interaction Frequency
0
3
6
9
2
4
6
8
Number of Individuals Where Hi-C Contact Called Significant
B
Number of Individuals a Hi-C Hit is Discovered in Relative to Variance
Variance in Interaction Frequency
0.00
0.25
0.50
0.75
1.00
2
4
6
8
Number of Individuals Where Hi-C Contact Called Significant
