# Supplementary material for: Reorganization of 3D genome structure may contribute to gene regulatory evolution in primates
Source: PLoS Genet. 2019 Jul 19;15(7):e1008278. doi: 10.1371/journal.pgen.1008278 (PMC6668850; doi:10.1371/journal.pgen.1008278)
Supplement: S8 Fig — (A) Histogram of log2(observed/expected) HOMER-normalized interaction frequencies in all four human samples used in this study, after applying pairwise cyclic loess normalization with limma [63]. (B) Same as A, but in chimpanzees. (PDF) [file pgen.1008278.s008.pdf]

A

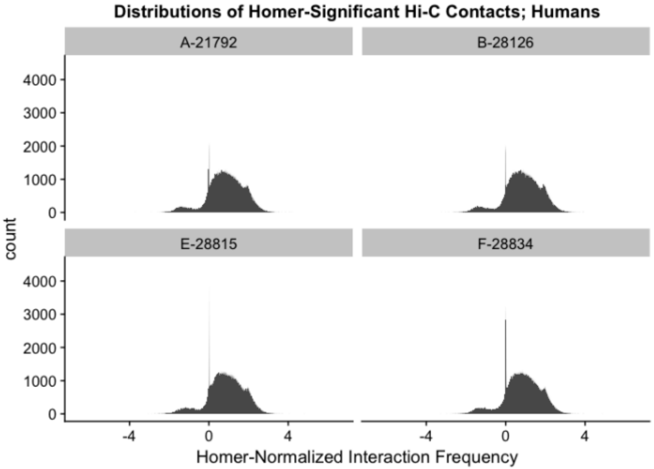

Distributions of Homer-Significant Hi-C Contacts; Humans
A-21792
B-28126
E-28815
F-28834
count
4000
3000
2000
1000
0
-4
0
4
Homer-Normalized Interaction Frequency

B

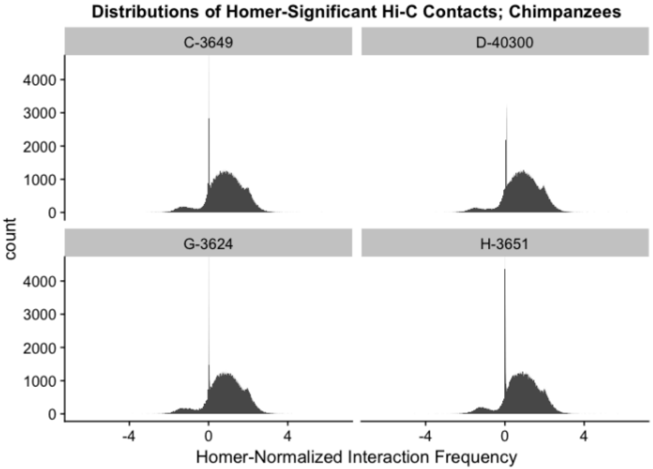

Distributions of Homer-Significant Hi-C Contacts; Chimpanzees
C-3649
D-40300
G-3624
H-3651
count
4000
3000
2000
1000
0
-4
0
4
Homer-Normalized Interaction Frequency
