# Supplementary material for: Reorganization of 3D genome structure may contribute to gene regulatory evolution in primates
Source: PLoS Genet. 2019 Jul 19;15(7):e1008278. doi: 10.1371/journal.pgen.1008278 (PMC6668850; doi:10.1371/journal.pgen.1008278)
Supplement: S12 Fig — (A) Across different resolutions (x-axis), we plotted the number of shared and species-specific domains (y-axis) identified with Arrowhead [62] on Juicer VC-normalized Hi-C maps from each individual. We called domain conservation here based on the method of Rao et al. [45] (highly similar results were observed with our 90% reciprocal overlap method, described in the text and available in the github repository associated with the paper). Domain count values represent the average interspecies sharing across all individuals, with no filtering for domain robustness (that is, assessing all domains discovered and orthologously mappable). Under this analysis paradigm we observe relatively low sharing across species (~60% at 10kb). (B) Same as A, but this time, only considering TADs that were found across all 4 individuals within either one of the species (fixed TADs). Restricting to this subset increases the percentage of conservation to 78%, although the set of TADs being examined is much smaller. (C) Same as A, but for boundaries instead of domains. Boundaries were defined as 15kb flanking regions at the edges of inferred Arrowhead domains. Because the TADs called by Arrowhead are nested, we merged boundaries here to obtain unique genomic intervals, rather than counting boundaries repeatedly. We then considered boundaries shared between individuals if they had any overlap. (D) Same as B, but for boundaries instead of domains (i.e. considering only boundaries fixed within species). Here, the highest estimate of conservation we obtain is 83% of boundaries conserved across species at 10kb resolution. (E) Unsupervised hierarchical clustering of the pairwise proportions of shared TADs between all individuals in our study at a variety of resolutions, using the Rao et al. [45] methodology for calling conservation. The first letter in the labels demarcates the species (H for human and C for chimpanzee), and the following symbols indicate sex (male, M or female, F) and batch (1 o [file pgen.1008278.s012.pdf]

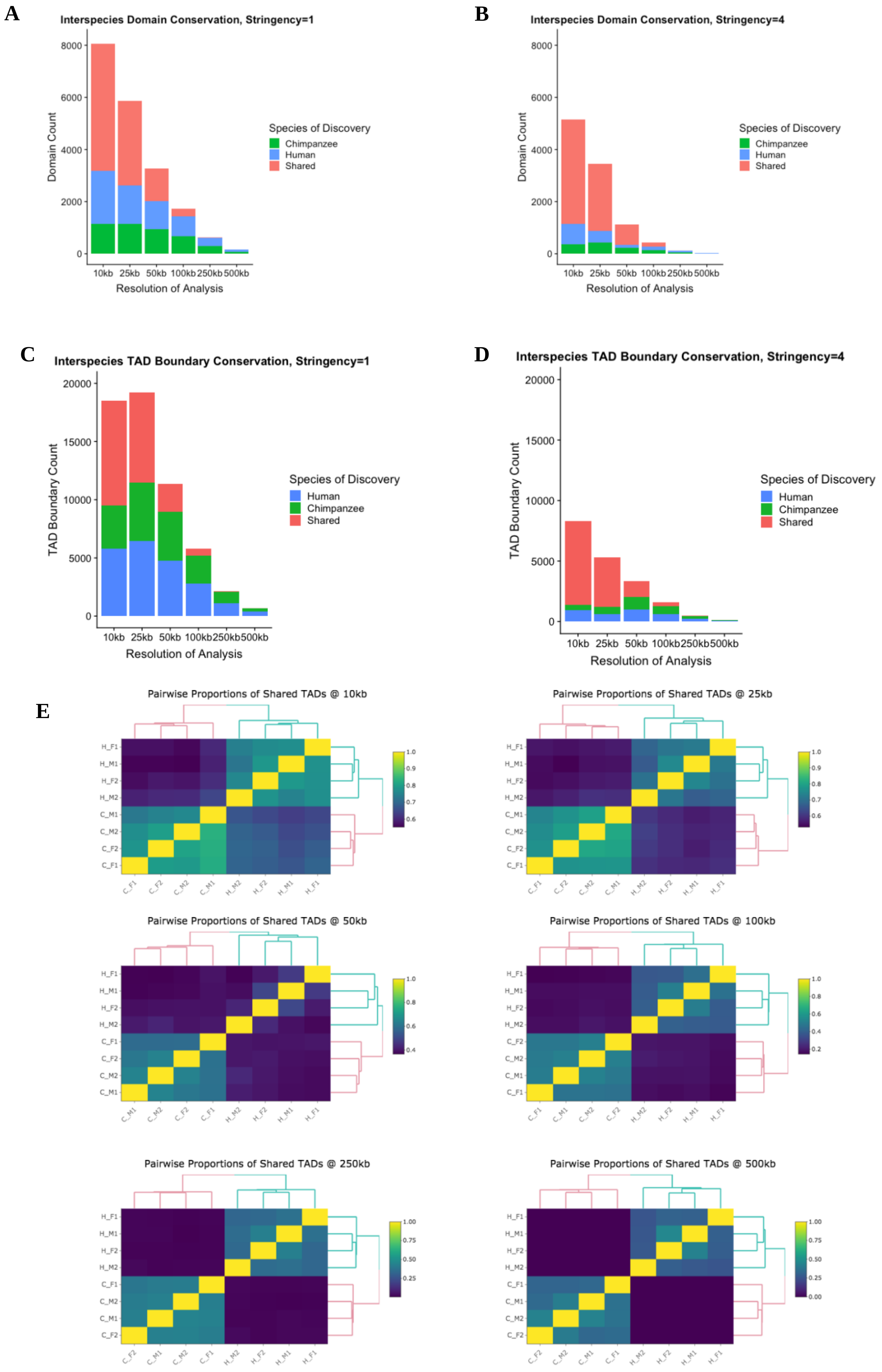
A
Interspecies Domain Conservation, Stringency=1
Domain Count
Resolution of Analysis
10kb 25kb 50kb 100kb 250kb 500kb
Species of Discovery
Chimpanzee
Human
Shared
B
Interspecies Domain Conservation, Stringency=4
Domain Count
Resolution of Analysis
Species of Discovery
Chimpanzee
Human
Shared
C
Interspecies TAD Boundary Conservation, Stringency=1
TAD Boundary Count
Resolution of Analysis
Species of Discovery
Human
Chimpanzee
Shared
D
Interspecies TAD Boundary Conservation, Stringency=4
TAD Boundary Count
Resolution of Analysis
Species of Discovery
Human
Chimpanzee
Shared
E
Pairwise Proportions of Shared TADs @ 10kb
Pairwise Proportions of Shared TADs @ 25kb
Pairwise Proportions of Shared TADs @ 50kb
Pairwise Proportions of Shared TADs @ 100kb
Pairwise Proportions of Shared TADs @ 250kb
Pairwise Proportions of Shared TADs @ 500kb

F

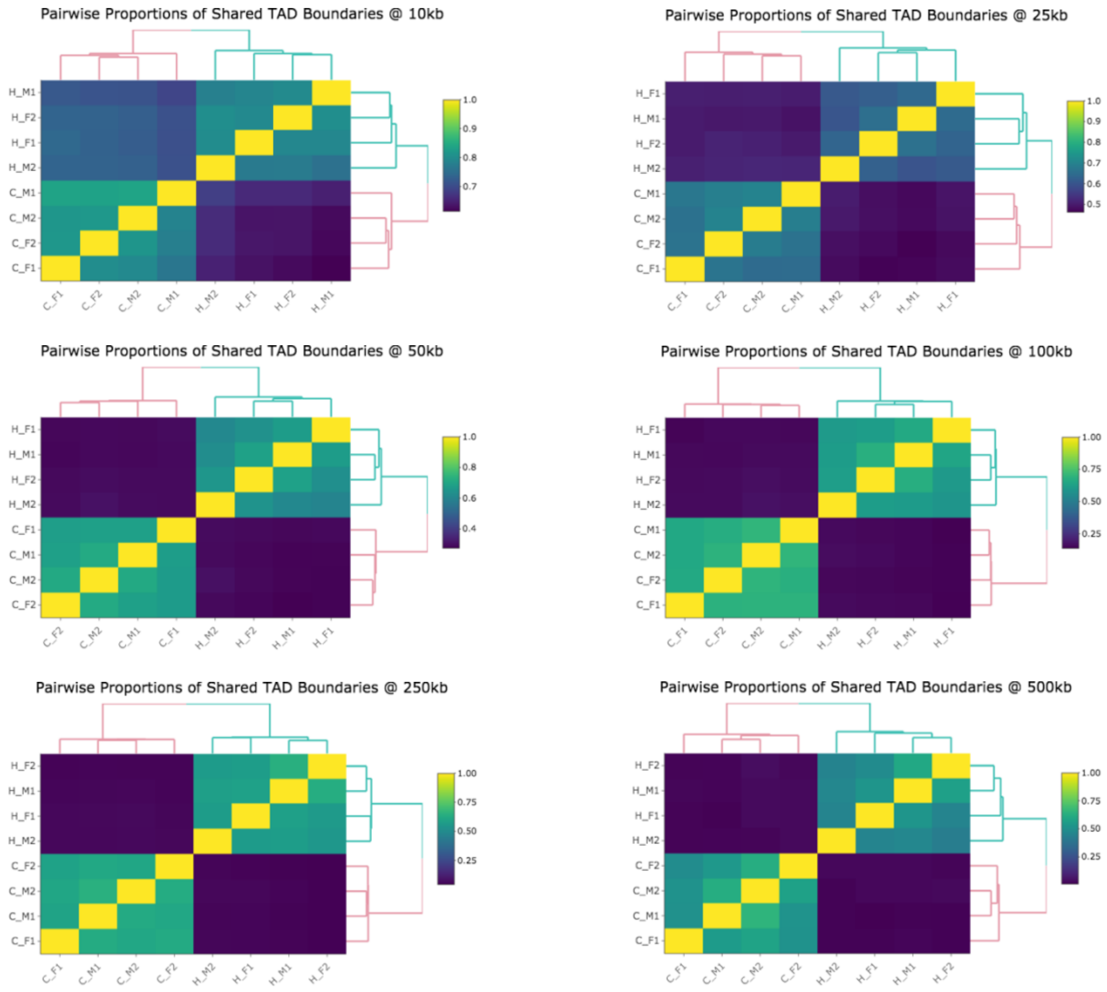
Pairwise Proportions of Shared TAD Boundaries @ 10kb
Pairwise Proportions of Shared TAD Boundaries @ 25kb
Pairwise Proportions of Shared TAD Boundaries @ 50kb
Pairwise Proportions of Shared TAD Boundaries @ 100kb
Pairwise Proportions of Shared TAD Boundaries @ 250kb
Pairwise Proportions of Shared TAD Boundaries @ 500kb
