# Supplementary material for: Reorganization of 3D genome structure may contribute to gene regulatory evolution in primates
Source: PLoS Genet. 2019 Jul 19;15(7):e1008278. doi: 10.1371/journal.pgen.1008278 (PMC6668850; doi:10.1371/journal.pgen.1008278)
Supplement: S17 Fig — (A) Histogram showing the number of Hi-C loci (y-axis) assigned to each chromHMM annotation (x-axis) using maximum base pair overlap to assign each locus to a state. In the legend, “.” denotes that no annotation was found for a given bin. (TssA-Active TSS, TSSBiv-Bivalent/Poised TSS, BivFlnk-Flanking Bivalent TSS/Enh, EnhBiv-Bivalent Enhancer, ReprPC-Repressed PolyComb, ReprPCWk-Weak Repressed PolyComb, Quies-Quiescent/Low, TssAFlnk-Flanking Active TSS, TxFlnk-Transcription at gene 5’ and 3’, Tx-Strong transcription, TxWk-Weak transcription, EnhG-Genic Enhancers, Enh-Enhancers, ZNF/Rpts-ZNF genes and repeats, Het-Heterochromatin). (B) Same as A, only here, we assigned annotations after weighting chromHMM elements’ overlaps with Hi-C loci by the reciprocal of their mean overlap in all our loci. This approach increases the number of 10kb Hi-C bins that are assigned to chromHMM annotations associated with transcriptional and enhancer activity (i.e. TssA, TssBiv, TssAFlnk, EnhG, Enh). (PDF) [file pgen.1008278.s017.pdf]

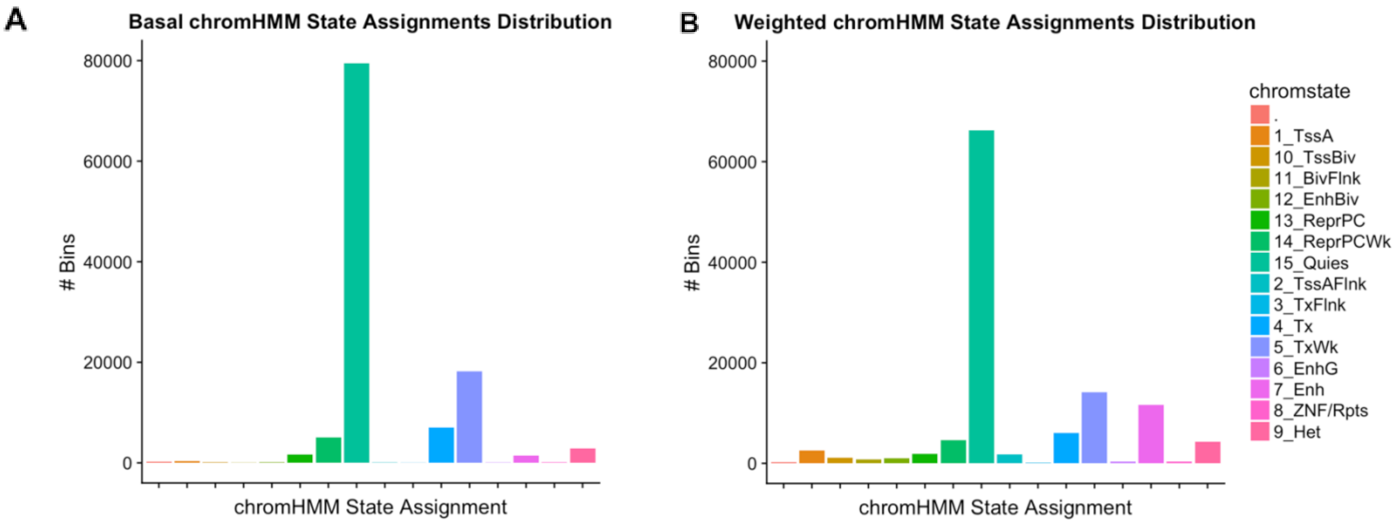
A
Basal chromHMM State Assignments Distribution
80000
60000
40000
20000
0
Bins
chromHMM State Assignment
B
Weighted chromHMM State Assignments Distribution
80000
60000
40000
20000
0
Bins
chromHMM State Assignment
chromstate
.
1_TssA
10_TssBiv
11_BivFlnk
12_EnhBiv
13_ReprPC
14_ReprPCWk
15_Quies
2_TssAFlnk
3_TxFlnk
4_Tx
5_TxWk
6_EnhG
7_Enh
8_ZNF/Rpts
9_Het
