# Supplementary material for: Reorganization of 3D genome structure may contribute to gene regulatory evolution in primates
Source: PLoS Genet. 2019 Jul 19;15(7):e1008278. doi: 10.1371/journal.pgen.1008278 (PMC6668850; doi:10.1371/journal.pgen.1008278)
Supplement: S19 Fig — (A) Hi-C loci that make contact with promoters of genes that are not differentially expressed (DE) across species are ranked in order of decreasing DC FDR (x-axis). The y-axis shows cumulative proportion of chromHMM annotation assignments for all Hi-C loci at the given FDR or lower. (TssA-Active TSS, TSSBiv-Bivalent/Poised TSS, BivFlnk-Flanking Bivalent TSS/Enh, EnhBiv-Bivalent Enhancer, ReprPC-Repressed PolyComb, ReprPCWk-Weak Repressed PolyComb, Quies-Quiescent/Low, TssAFlnk-Flanking Active TSS, TxFlnk-Transcription at gene 5’ and 3’, Tx-Strong transcription, TxWk-Weak transcription, EnhG-Genic Enhancers, Enh-Enhancers, ZNF/Rpts-ZNF genes and repeats, Het-Heterochromatin). (B) Same as A, but only considering Hi-C loci making contact with promoters of genes that are differentially expressed (DE). (PDF) [file pgen.1008278.s019.pdf]

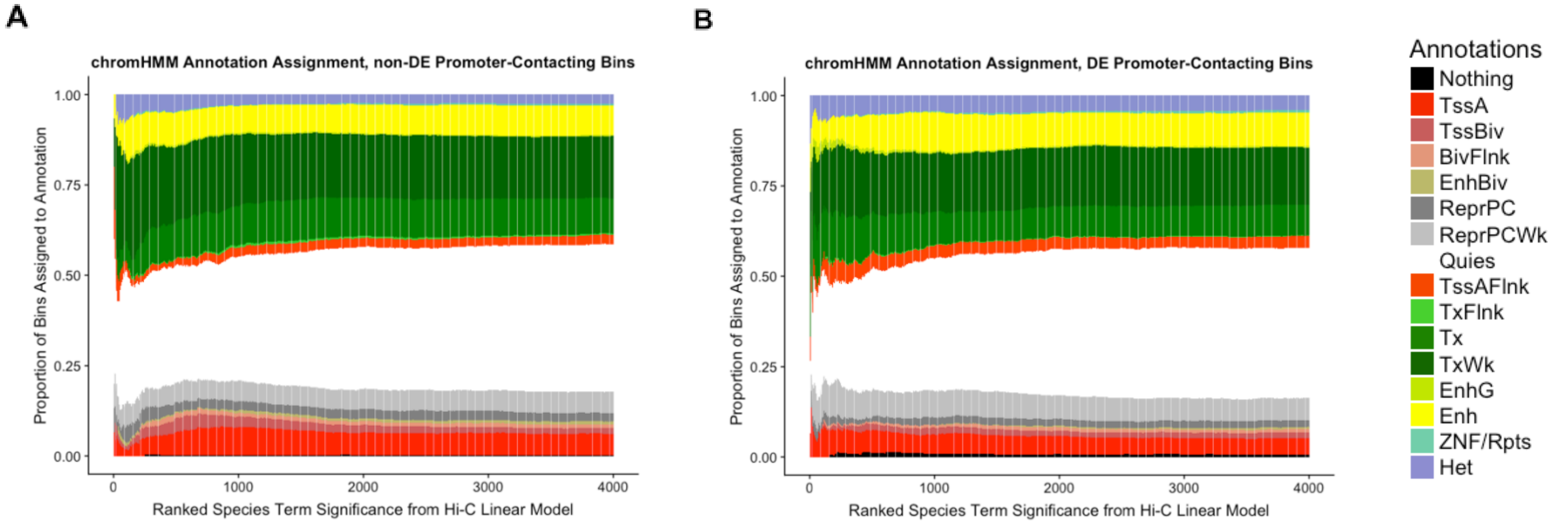

A
chromHMM Annotation Assignment, non-DE Promoter-Contacting Bins
Proportion of Bins Assigned to Annotation
1.00
0.75
0.50
0.25
0.00
0
1000
2000
3000
4000
Ranked Species Term Significance from Hi-C Linear Model
B
chromHMM Annotation Assignment, DE Promoter-Contacting Bins
Proportion of Bins Assigned to Annotation
1.00
0.75
0.50
0.25
0.00
0
1000
2000
3000
4000
Ranked Species Term Significance from Hi-C Linear Model
Annotations
Nothing
TssA
TssBiv
BivFlnk
EnhBiv
ReprPC
ReprPCWk
Quies
TssAFlnk
TxFlnk
Tx
TxWk
EnhG
Enh
ZNF/Rpts
Het
